# Comprehensive genome analysis of *Burkholderia contaminans* SK875, a quorum-sensing strain isolated from the swine

**DOI:** 10.1186/s13568-023-01537-8

**Published:** 2023-03-11

**Authors:** Eiseul Kim, Hae-In Jung, Si Hong Park, Hae-Yeong Kim, Soo-Ki Kim

**Affiliations:** 1 Institute of Life Sciences & Resources and Department of Food Science and Biotechnology, Kyung Hee University, Yongin, 17104 Korea; 2 Department of Animal Sciences and Technology, Konkuk University, Seoul, 05029 Korea; 3 Department of Food Science and Technology, Oregon State University, Corvallis, OR 97331 USA

**Keywords:** *Burkholderia contaminans*, Whole-genome sequencing, Comparative genomics, Quorum sensing

## Abstract

The *Burkholderia cepacia* complex (BCC) is a Gram-negative bacterial, including *Burkholderia contaminans* species. Although the plain *Burkholderia* is pervasive from taxonomic and genetic perspectives, a common characteristic is that they may use the quorum-sensing (QS) system. In our previous study, we generated the complete genome sequence of *Burkholderia contaminans* SK875 isolated from the respiratory tract. To our knowledge, this is the first study to report functional genomic features of *B*. *contaminans* SK875 for understanding the pathogenic characteristics. In addition, comparative genomic analysis for five *B*. *contaminans* genomes was performed to provide comprehensive information on the disease potential of *B*. *contaminans* species. Analysis of average nucleotide identity (ANI) showed that the genome has high similarity (> 96%) with other *B*. *contaminans* strains. Five *B. contaminans* genomes yielded a pangenome of 8832 coding genes, a core genome of 5452 genes, the accessory genome of 2128 genes, and a unique genome of 1252 genes. The 186 genes were specific to *B*. *contaminans* SK875, including toxin higB-2, oxygen-dependent choline dehydrogenase, and hypothetical proteins. Genotypic analysis of the antimicrobial resistance of *B*. *contaminans* SK875 verified resistance to tetracycline, fluoroquinolone, and aminoglycoside. Compared with the virulence factor database, we identified 79 promising virulence genes such as adhesion system, invasions, antiphagocytic, and secretion systems. Moreover, 45 genes of 57 QS-related genes that were identified in *B. contaminans* SK875 indicated high sequence homology with other *B. contaminans* strains. Our results will help to gain insight into virulence, antibiotic resistance, and quorum sensing for *B. contaminans* species.

**Supplementary Information:**

The online version contains supplementary material available at 10.1186/s13568-023-01537-8.

## Keypoints

- We first report the genome characteristics of B. contaminans SK875.
- The B. contaminans SK875 genome carries virulence and quorum sensing-related genes.
- The SK875 has 186 unique genes, such as toxin higB, betA, and hypothetical proteins

**Supplementary Information:**

The online version contains supplementary material available at 10.1186/s13568-023-01537-8.

## Introduction

*Burkholderia cepacia* complex (BCC) is a group of genetically distinct, phenotypically similar Gram-negative bacteria (Mahenthiralingam et al. 2005). The prevalence of BCC species varies geographically. *Burkholderia cenocepacia* is the most predominant species in North American cystic fibrosis (CF) centers, while *Burkholderia multivoran*s are the most common species in European CF centers (Govan et al. 2007; Sousa et al. 2011). Nevertheless, outbreaks caused by other BCC species have occurred worldwide (Sousa et al. 2011). The BCC is a group of 20 closely-related bacterial species that share up to 78% of their genes (Holden et al. 2009). These bacteria have large genomes ranging from 7 to more than 9 Mb, generally arranged in three chromosomes and large plasmids (Ussery et al. 2009; Holden et al. 2009). Recently, these bacteria were recognized as a threat to hospitalized patients being affected by other diseases, notably oncological ones (Wong and Evans 2017). The BCC species have been extensively studied and differentiated regarding their virulence and transmissibility (McClean and Callaghan 2009). Since the precise mechanisms by which they spread among patients or which of the natural BCC reservoirs pose the greatest risk to cystic fibrosis patients are still unknown, the BCC continues to be a very problematic CF pathogen (Mahenthiralingam et al. 2005). Currently, 17 species have been officially designated as members of the complex, including *Burkholderia contaminans* (Rose et al. 2009). Nevertheless, because not all species in this group of closely-related species are equally transmissible and dispersed throughout the environment, it follows that there should be differences in the genome. Researchers face challenges that *Burkholderia* species are extensive from taxonomic and genetic perspectives.

Despite the clear genetic diversity within the *Burkholderia* genus, a common characteristic is that they may use quorum sensing (QS) as a part of their colonization and invasion strategies (Choudhary et al. 2013). Processes controlled by QS include activating bacterial defense mechanisms, including the synchronized production of virulence factors and biofilm (Parsek and Greenberg 2005). QS systems appear crucial in governing overall colonization and niche invasion (Ng and Bassler 2009). These reactions are triggered by the extracellular concentration of bacterially produced and secreted small soluble autoinducer signal molecules (Ng and Bassler 2009). Interestingly, bacteria usually do not rely on a single signal molecule; however, within a single organism, different QS systems may operate either in parallel or hierarchically (Papenfort and Bassler 2016).

Gram-negative bacteria’s most prevalent QS system relies on synthesizing and reacting to N-acylated homoserine lactones (AHLs). Production is catalyzed by an AHL synthase belonging to the LuxI-family of proteins (Papenfort and Bassler 2016). The LuxIR homolog CepIR was the first QS system to be identified in a *B. cenocepacia* strain (Jacobs et al. 2008; McClean and Callaghan 2009; Vanlaere et al. 2009). The CepIR system relies on the AHL synthase CepI and the transcriptional regulator CepR that binds explicitly to AHL, becoming active (Gotschlich et al. 2001). Additionally, the QS system based on the fatty acid molecule cis-2-dodecenoic acid as the signaling molecule was identified for the first time in *B. cenocepacia* J2315 and was named *Burkholderia* diffusible signal factor (BDSF) (Deng et al. 2011). The RpfFR QS system, which is highly conserved within BCC, uses this molecule as its signaling molecule. The RpfFR system relies on the biosynthesis of BDSF by the bifunctional crotonase RpfF and the BDSF receptor protein RpfR containing PAS-GGDEF-EAL domains (Yang et al. 2017). The signaling molecule BDSF binds to RpfR, stimulating the cyclic dimeric guanosine monophosphate (c-di-GMP) phosphodiesterase activity of the protein, thus, lowering the intracellular c-di-GMP levels (Deng et al. 2012; Schmid et al. 2012). Many genes positively regulated by RpfFR are also controlled by the CepIR QS system (Suppiger et al. 2013). Although their roles appear parallel, it was discovered that each QS system’s contribution to the regulation of target genes was variable.

Therefore, we should understand the complicated QS systems in *Burkholderia*, particularly the genetic basis upon which they operate. Here, we analyzed the entire genome of *B. contaminans* SK875, which was initially isolated from the respiratory tract of swine, to identify antibiotic resistance and virulence loci. Additionally, we analyzed the phylogenetic relatedness of SK875 to 144 other *Burkholderia* species with other four *B. contaminans* species, including plant pathogens, CF opportunists, plant growth-promoting strains, and other soil isolates. The findings provide essential information for evaluating *Burkholderia* species’ virulence according to their secondary metabolite production and virulence markers. We compared two QS systems using bioinformatic analysis on *B. contaminans* species genome, and QS-related genes screened in the previous study were mapped on the B. *contaminans* SK875 genome.

## Materials and methods

### Complete genome sequence of B. *contaminans* SK875

*B*. *contaminans* SK875 were initially isolated from the respiratory tract of swine (Jung et al. 2017), but it was not the causative agent of the respiratory disease of the swine. The detailed information has been reported previously (Jung et al. 2020). Briefly, the genomic DNA of the B. *contaminans* SK875 strain was sequenced using the Illumina HiSeq 2000 platform (Illumina, San Diego, CA, USA) and PacBio RS II system (Pacific Bioscience, Menlo Park, CA, USA). De novo assembly of *B. contaminans* SK875 was performed using the hierarchical genome assembly process v.2.3. Finally, three chromosomes and one plasmid were produced for the *B. contaminans* SK875 strain. Rapid Prokaryotic Genome Annotation was used for the genome annotation (Prokka) v.1.10 (Seemann 2014).

### In silico taxonomy identification

For the analysis of phylogeny comparison, 80 complete genome sequences of *Burkholderia* species, including 3 *B. ambifaria*, 20 *B. cenocepacia*, 13 *B. cepacia*, 5 *B. contaminans*, 2 *B. dolosa*, 3 *B. lata*, 15 *B. multivorans*, 2 *B. anthina*, 2 *B. pyrrocinia*, 2 *B. stabilis*, 6 *B. ubonensis*, and 7 *B. vietnamiensis*, were collected from the National Center for Biotechnology Information (NCBI) (Additional file 1: Table S1).

The average nucleotide identity (ANI) was conducted using the JSpeciesWS (Richter et al. 2016). The value was determined using the ANIb algorithm with the constructed sequences as input. With the aid of the bacterial pangenome analysis (BPGA) tool, an evolutionary study based on binary pan-matrix (binary gene presence/absence matrix) and concatenated core gene alignments was conducted (Chaudhari et al. 2016). Furthermore, the pan and core genomes were aligned using MUSCLE, and a neighbor-joining tree was constructed. The tree was visualized using iTOL (Letunic and Bork 2016).

### Pangenome analysis of *B. contaminans*

Five complete genome sequences of *B. contaminans*, including SK875, MS14, ZCC, XL73, and FL-1-2-30-S1-D0, were used for the pangenome analysis. Phylogeny analysis based on the pangenome of five *B. contaminans* genomes was performed using the Anvi’o pangenome pipeline version 6.0 (Eren et al. 2015). The “anvi-gen-genomes-storage” tool was used to create genome storage databases containing information about the genomes being studied for phylogeny research. Using the “anvi-pangenome” tool, the produced genome storage was submitted to pan-genomic analysis. Subsequently, the pangenome was shown using the “anvi-display-pan” program and constructed following gene cluster frequencies.

Pangenome analysis was conducted to determine genomes’ accessory, core, and unique genes. The BPGA tool was used as the pipeline for pangenome analysis of the five *B. contaminans* genomes. For pangenome analysis, protein sequences of each whole-genome sequence were used. All analyses were performed using default parameters with an identity cut-off value = 0.5. Using the pangenome functional analysis module in BPGA, the orthologous protein clusters were allocated to the clusters of orthologous groups (COGs) and Kyoto Encyclopedia of Genes and Genomes (KEGG) categories.

### Antimicrobial and virulence genes

Resistance gene identifier (RGI) software v.5.1.1 (Alcock et al. 2020) was used to detect intrinsic or acquired antimicrobial genes in five *B. contaminans* genomes. For this purpose, FASTA assembled files were used as inputs and identified with a comprehensive antibiotic resistance database (CARD) 3.1.1 using DIAMOND. Antimicrobial genes were detected using criteria of perfect and strict hits only, high sequence quality, and coverage, excluding nudging of ≥ 95% identity loose hits to severe. The possible virulence factors in the five *B. contaminans* genomes were identified by generating orthologous groups with VFanalyzer and then comparing them to reference genomes from the virulence factors database (VFDB) (Liu et al. 2019).

### Identification of CRISPR region, phage, and secretion system

Prophage elements in the five *B. contaminans* genomes were searched using the phase search tool enhanced release (PHASTER) tool (Arndt et al. 2016). Clustered, regularly interspaced short palindromic repeats (CRISPR) loci were identified using the CRISPRCasFinder (version CRISPR-Cas +  + 1.1.2.) (Couvin et al. 2018).

The pathway for the QS system in five *B. contaminans* genomes was explored using the KEGG database (https://www.kegg.jp/). Genes associated with the CepI/CepR and RpfF/RpfR systems were evaluated by the BlastKOALA search tool v.2.2 in the KEGG database. Sequences were compared using Easyfig version 2.2.5 (BLASTn, default setting).

## Results

### General genome feature

*B. contaminans* SK875 strain has published genome sequence in the public database without being deposited in the publicly accessible culture collection. The entire genome of *B. contaminans* SK875 included three circular chromosomes of 1528467 to 3,618903 bp with GC contents of 65.8 to 66.4% and one plasmid of 200961 bp with a GC content of 61.7%. The entire genome harbored 7625 proteins, 18 rRNAs, and 83 tRNAs. The genomic characteristics of *B. contaminans* SK875 and the other four *B. contaminans* strains are presented in Table 1. The average complete genome length of the five *B. contaminans* was 8.59 Mb ranging from 8.17 to 9.00 Mb, and the moderate GC content was 66.31% ranging from 66.06 to 66.53%. Among these strains, *B. contaminans* FL-1-2-30-S1-D0 strain indicated high GC content (66.53%), and the *B. contaminans* ZCC strain demonstrated lower GC content (66.06%).

**Table 1:** Comparison of genomic features of *B. contaminans* SK875 and other strains

Strain	Size (Mb)	GC%	CDS	rRNA	tRNA	Source
*B. contaminans* MS14	8.50925	66.40	7494	15	69	Soil
*B. contaminans* FL-1-2-30-S1-D0	8.17078	66.53	7138	18	69	Unknown
*B. contaminans* ZCC	9.00224	66.06	7724	18	69	Soil
*B. contaminans* SK875	8.59605	66.30	7564	18	83	Respiratory tract of pig
*B. contaminans* XL73	8.65686	66.27	7565	18	69	Cucumber rhizosphere

In the *B. contaminans* SK875 genome, a high proportion of genes in COG functional categories were allocated to the common function prediction only (R, 14.15%), amino acid transport and metabolism (E, 11.06%), and transcription (K, 10.87%) (Additional file 1: Table S2). Because of mapping the KEGG pathway database to the *B. contaminans* SK875 genome, coding genes were allocated to 43 functional categories and 235 pathways, mainly functioning in the ABC transporters (ko:02010), the biosynthesis of amino acids (ko:01230), carbon metabolism (ko:01200), and two-component system (ko:02020) (Additional file 1: Table S3).

Previous studies found that *B. multivorans* strains were augmented in genes associated with translation (J) and replication (L). In contrast, genes related to transcription (K) were deprived and thus had lower adaptability of them to varying environments (Peeters et al. 2017). Furthermore, numerous studies have shown that the proportion of genes engaged in translation and replication negatively affect genome size, whereas transcription positively correlates with genome size (Konstantinidis and Tiedje 2004; Peeters et al. 2017). *B. contaminans*, including SK875 strain, have a larger genome size than other *Burkholderia* species, including *B. multivorans*, and harbor a relatively high proportion of genes involved in transcription, implying that they can adapt well to different environments.

### Taxonomic position of the *B. contaminans* SK875 strain

With the advancement in sequencing technology, many whole-genome sequences are now available in publicly accessible databases, and genome analysis can be used to identify a novel strain (Liang et al. 2019). Bacterial species determination and categorization using ANI analysis provides a higher resolution than other identification techniques and can prevent bias due to sequence selection (Han et al. 2016). Our study used ANI, an in silico method for pairwise comparisons of all sequences, to categorize *B*. *contaminans* SK875 strain species. ANI analysis is conducted to study species’ genetic and evolutionary distance, and values of > 95% are the accepted cut-off threshold for species delineation (Chua et al. 2019).

**Fig. 1:**
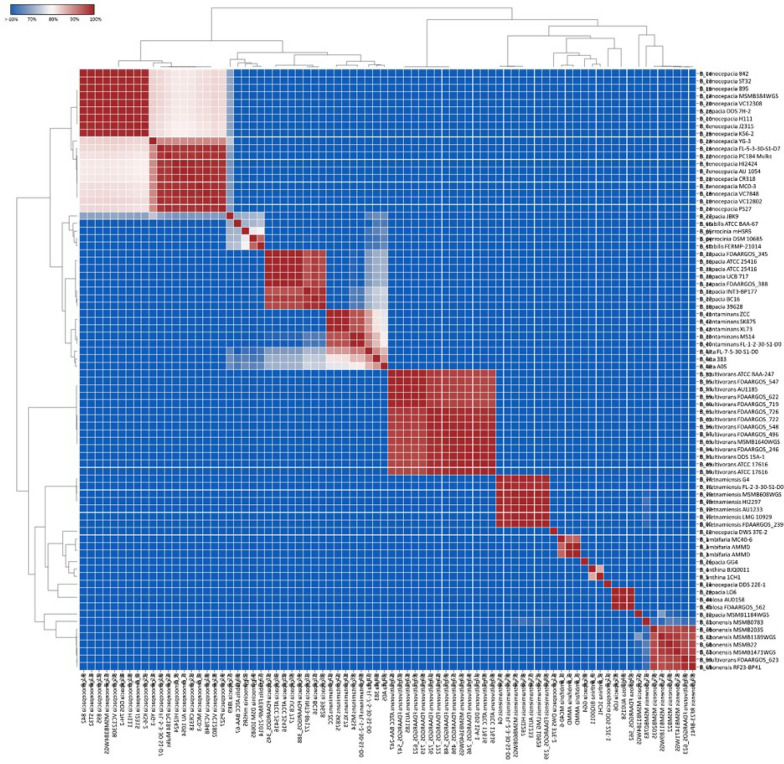
Clustering of 80 *Burkholderia* genome sequences on the basis of average nucleotide identity (ANI). Heatmap shows the level of sequence similarity on the scale 60 to 100 where > 60% similarity is depicted in blue and 100% similarity is depicted in red

Based on the ANI calculation, *B. contaminans* were most closely associated with *B. lata*. *B. contaminans* SK875 strain shared 96.14% to 99.99% sequence similarities with five *B. contaminans* strains (Fig. 1). *B. contaminans* SK875 strain showed the closest relationship with the ZCC strain (99.99% identity) among the *B. contaminans* genomes. *B. contaminans* ZCC strain was first isolated from mining soil and is a promising cadmium-resistant strain that enhances the growth of soybeans in the presence of cadmium (You et al. 2021). *B. contaminans* SK875 strain shared 84.99 to 94.57% low sequence identities with other *Burkholderia* species.

**Fig. 2:**
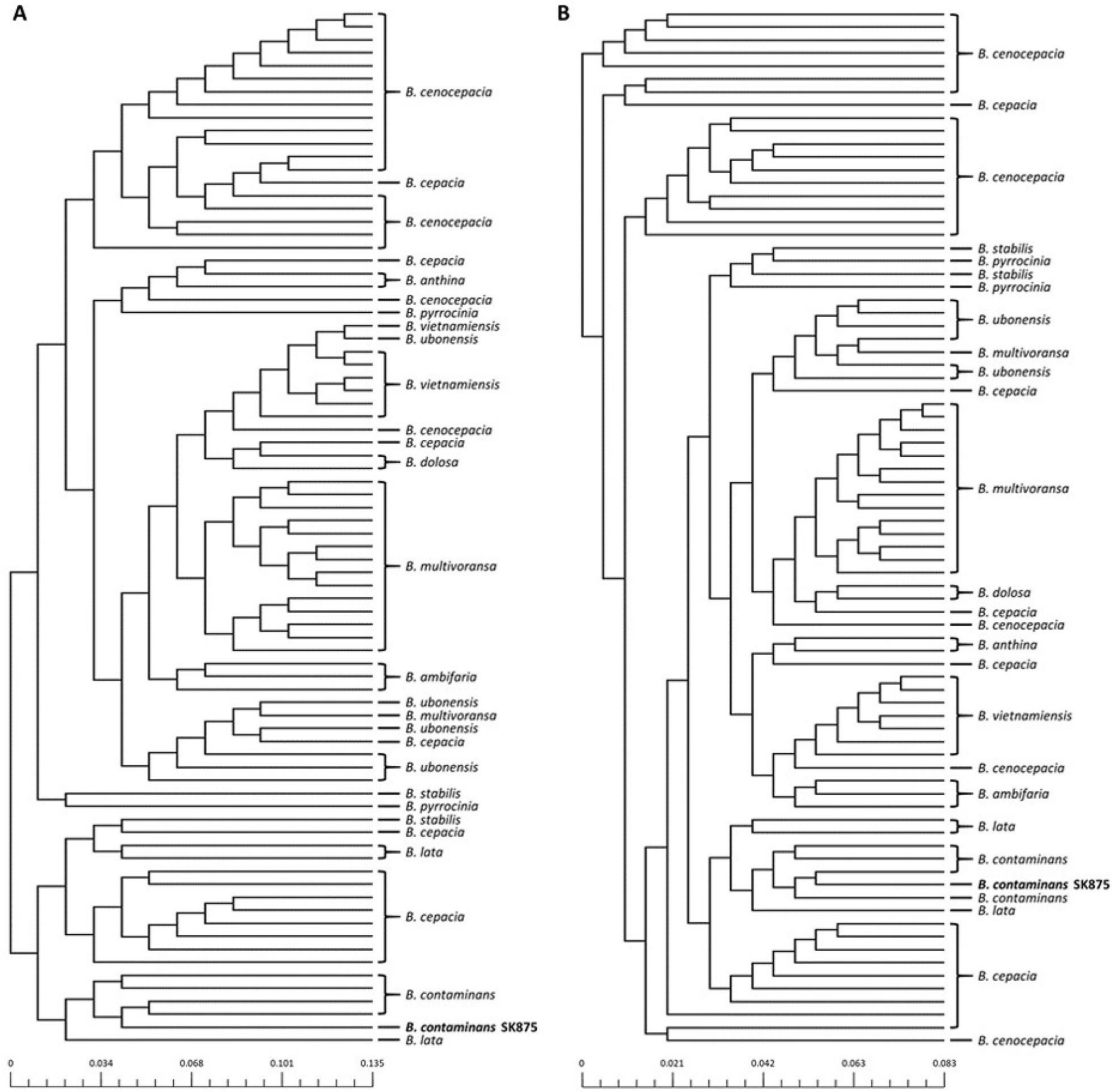
Phylogenetic tree for 80 complete *Burkholderia* genome sequences based on (**A**) binary pan-matrix and (**B**) concatenated core genes

A phylogenetic comparison was performed using pan- and core-genomes to infer the phylogenetic relationship between *B*. *contaminans* SK875 and other *Burkholderia* species with completed genome sequences. The pan phylogenetic tree was constructed based on a binary pan-matrix (presence or absence of genes) (Chaudhari et al. 2016). All genomes showed the same phylogeny, depending on the species. The *B. contaminans* SK875 strain was clustered with *B. contaminans* strains (Fig. 2A). The conclusions of the phylogenetic tree based on the core genome were congruent with the findings of the pangenome analysis (Fig. 2B). In the phylogenetic tree according to the pan- and core-genomes, *B. contaminans* SK875 strain was categorized as *B. contaminans* and demonstrated the closest relationship with XL73 and ZCC strains.

### Pangenome analysis of *B. contaminans*

**Fig. 3:**
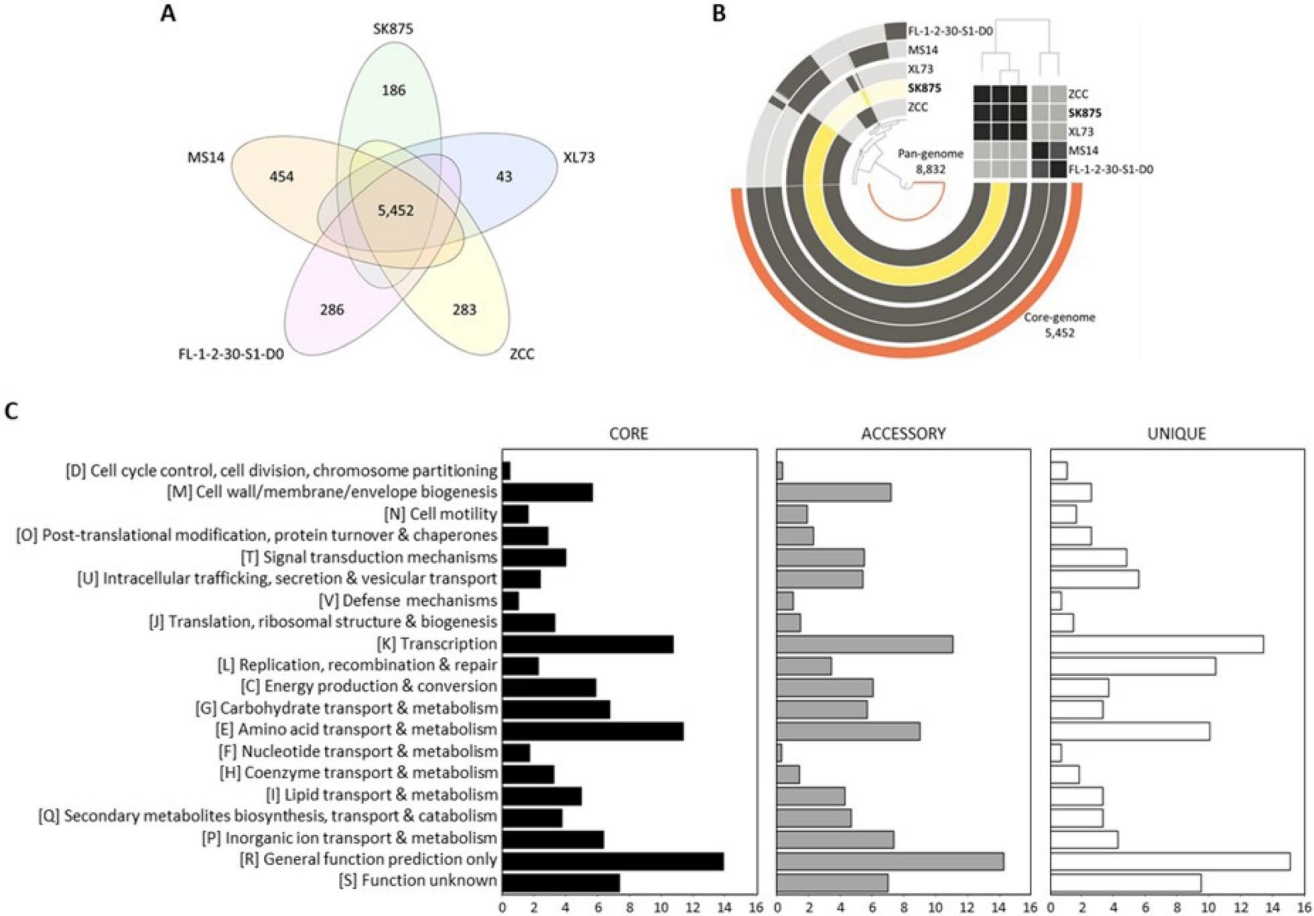
Pan-genome distribution of five *B. contaminans* genomes. **A** The number of core, accessory, and unique genes among *B. contaminans* strains. **B** Phylogenetic tree based on pan-genome distribution. Each ring represents one genome, and the dark and light colors of the ring indicate the presence and absence of genes, respectively. The ANI values on the right are represented by heatmap determined at low (gray) and high similarity (black). **C** The number of pan genes assigned in the COG database

Pangenome analysis can be used to investigate the core, auxiliary, and unique genes in pathogenic bacterial genomes (Liang et al. 2019). The core gene is frequently found in all strains, the accessory gene is present in two or more strains, and the unique gene is unique to individual members of a species (Bosi et al. 2016; Li et al. 2020). Five *B. contaminans* genomes yielded a pangenome size of 8832 genes; of these, the core genome is composed of 5452 genes (61.73%), and the accessory genome is composed of 2128 genes (24.09%). The unique genome is composed of 1252 genes (14.18%) (Fig. 3). Mainly, the core and individual genes of the *B. contaminans* SK875 strain consist of 5452 (71.50%) and 186 (2.44%) out of a total of 7625 orthologous genes.

The COG database functional annotation of pan genes revealed a heterogeneous distribution of categories across three pangenome sets. The 8832 pan gene clusters could be allocated in 20 COG categories (Fig. 3). Functional analysis showed that most core gene and accessory gene families were related to standard function prediction only (R, 13.92% and 14.26%), amino acid transport and metabolism (E, 11.40% and 9%), and transcription (K, 10.75% and 11.07%). The unique genome was primarily discovered in the general function prediction only (R, 15.11%), transcription (K, 13.43%), and replication, recombination, and repair (L, 10.45%).

Functional analysis by COGs in five *B. contaminans* genomes showed that most core and accessory gene families were related to metabolism, and the unique gene families were associated with information storage and processing. This finding is similar to a previous study that the individual genome of *Elizabethkingia*, pathogenic bacteria, had the highest proportion of genes in information storage and processing associated with intercellular survival. However, the exact reason for the high proportion of genes with this function in a unique gene is unclear (Liang et al. 2019). Compared with the previous studies, it was confirmed that *B. contaminans* contained less gene involvement in defense mechanisms than the *B. cenocepacia* and *B. multivorans* genomes (Peeters et al. 2017). This result implies that *B. contaminans* is generally less virulent than other *Burkholderia* species.

The number of strain-specific genes ranged from 43 genes only discovered in the MS14 strain to 454 genes unique to the XL73 strain. *B. contaminans* SK875 possessed 186 unique genes, including 22 proteins with known functions and 164 proteins with unknown functions of hypothetical proteins (Additional file 1: Table S4). COG analysis was used to further study the functioning of proteins encoded by unique genes in the SK875 genome. The SK875 genome contains 186 distinct genes, 32 of which have been classified into 14 COG functional categories (Additional file 1: Table S5). Functional analysis revealed that unique gene families were related to replication, recombination, and repair (L), transcription (K), energy production and conversion (C), amino acid transport and metabolism (E), and secondary metabolites biosynthesis, transport, and catabolism (Q). The classes general function prediction only (R) and function unknown (S) were represented in the unique genome, indicating that additional effort will be necessary to discover the potential functions of unique genes.

### Mobile gene elements in *B. contaminans*

Horizontal gene transfer mediated by transposons, phages, or plasmids is considered a mechanism responsible for the broad distribution of biodegradative pathways in pathogenic bacterial strains (Wu et al. 2019). Pathogenic bacterial strains have evolved both offensive (i.e., toxin and secretion systems) and defensive (i.e., phase variation and serum resistance) pathways (Yu et al. 2006). Prophages were found using the PHASTER across five *B*. *contaminans* genomes. Overall, 14 prophages were identified (Additional file 1: Table S6). There was only one intact prophage (score > 90), while two were questionable (score 70–90), and 11 were incomplete phages (score < 70). Each strain had an average of two or three prophages. Except for the SK875 strain, all strains included three prophages, while the SK875 strain contained two prophages. Two prophages were present in chromosome 1 of the SK875 genome. The prophage 1 region extended from 270930 to 279358 bp with a GC content of 61.69% and contained eight coding genes. The prophage 2 region extended from 2586105 to 2592869 bp with a GC content of 66.03% and contained eight coding genes. Prophage 1 and 2 regions demonstrated high homologous with phi92 found in *Enterobacteria* and RP12 found in *Ralstonia*, respectively.

There were often two or three prophages for each strain. All strains had three prophages, except for the SK875 strain, which had only two (Hu et al. 2019). It has been reported that CRISPR may be involved in the improvement of pathogenicity and regulation of virulence gene expression (Hu et al. 2019). A comparative analysis of five *B. contaminans* strains showed that most genomes contained more than one CRISPR (Additional file 1: Table S7). All strains had one to four CRISPR motifs without the cas operon. This is consistent with previous studies showing that deletion events of cas proteins have occurred in many *Burkholderia* strains such as *B. glumae* and *B. gladioli* (Seo et al. 2015). The maximum number of CRISPR (n = 4) was identified in the XL73 strain, whereas only one was detected in the MS14 and ZCC strains. *B. contaminans* SK875 genome contained two CRISPR loci. CRISPR1 and CRISPR2 were located nucleotides at 2456771 to 2457221 bp with five spacers and 2981872 to 2982191 bp with four spacers, respectively. Our results propose that, like other pathogenic bacteria, the *B. contaminans* could initiate a defense mechanism against foreign gene invasion to maintain the stability of the genetic structure during evolution (Li et al. 2020). Moreover, the results indicated that most mobile gene elements were distributed on chromosomes within the *B. contaminans*.

### Virulence genes and antimicrobial resistance genes

Genomic analysis of virulence genes showed similar virulence profiles among the *B*. *contaminans* strains (Additional file 1: Table S8). The significant virulence genes found in all genomes were related to the adhesion, invasion, antiphagocytic, and secretion systems. B. *contaminans* SK875 possessed *boaA*, *boaB*, and Type IV* pili* genes related to adherence to the human intestinal cells. Genomes of five B. *contaminans* had many capsules of synthesis-related and flagella-related genes. The expression of diverse virulence factors controlled by the secretion system is critical for bacterial pathogenesis (Rutherford and Bassler 2012). For secretion systems, *Burkholderia* secretion apparatus (Bsa) type III secretion system (T3SS) and type VI secretion system (T6SS-I) were commonly found in five *B. contaminans* genomes. The effector proteins that elude the immune system and cause disease are delivered into host cells with the aid of the T3SS system (Kendall 2017). Within the *Burkholderia* family, there are unique effector proteins such as *bsaQ*, *bsaS*, and *bsaX* (Vander Broek and Stevens 2017). Of these, the *bsaQ* gene (SK875_B00413) was present in the *B*. *contaminans* SK875 genome. The effector protein is located on the chromosomal pathogenicity island in chromosome SK875-2, so it can be transmitted to other cells by horizontal gene transfer (Brown and Finlay 2011). The protein transport mechanism from Gram-negative bacteria to eukaryotic cells was described as T6SS (Yang et al. 2018). These results suggest that five *B. contaminans* genomes, similar to other *Burkholderia* species, have the basic pathogenic mechanisms necessary to cause different infections regardless of the isolation environment (Peeters et al. 2017).

Antibiotic resistance is one of the standard features of the *Burkholderia* genus. Possessing multiple efflux pumps can increase the viability of bacterial strains in various ecological niches (Deng et al. 2016). The CARD and RGI databases were used to forecast the antibiotic resistance genes that five *B. contaminans* genomes contained. At least five or more antibiotic resistance genes were present in every genome (Additional file 1: Table S9). The resistance genes detected in five *B. contaminans* strains were similar. All strains had genes associated with aminoglycosides, fluoroquinolone/tetracycline, and tetracycline. *B. contaminans* SK875 strain showed seven antibiotic resistance genes conferring resistance to three various groups of antibiotics, including tetracycline (*tet(D)*), fluoroquinolone/tetracycline (*afeF*), and aminoglycoside (*amrA*). Overall, to other *Burkholderia* species, all strains of *B. contaminans* possess multiple antibiotic resistance and virulence-related genes (Deng et al. 2016). Therefore, our findings may provide genetic data to understand pathogenic *Burkholderia* had multiple antibiotic resistance and virulence mechanisms to survive and adapt to various environments.

### Quorum sensing-related genes

**Fig. 4:**
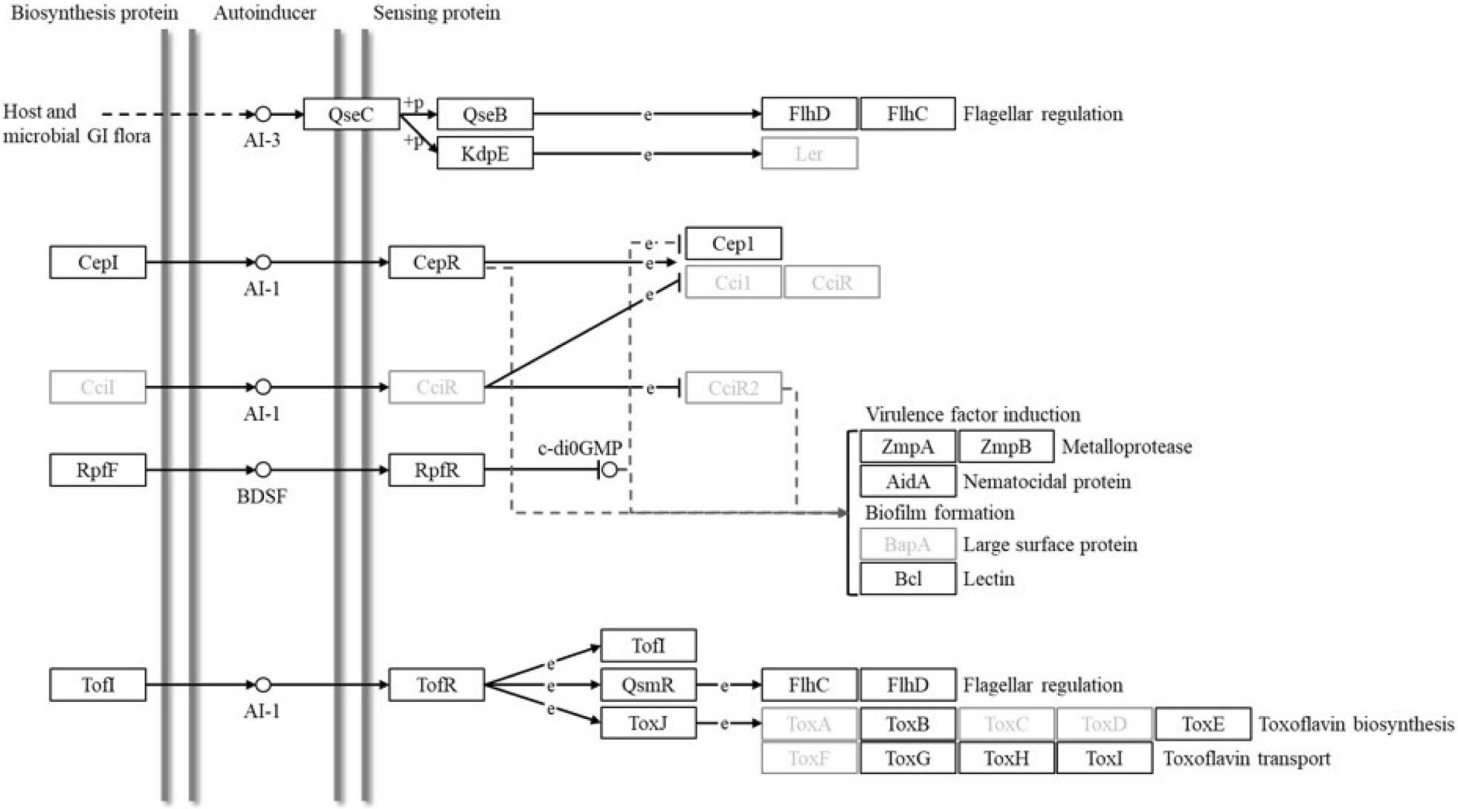
The Kyoto Encyclopedia of Genes and Genomes (KEGG) pathway for quorum sensing in *B. contaminans* SK875. The black box proteins and gray box proteins indicate the presence and absence of proteins, respectively

The *B*. *contaminans* SK875 genome was mapped using the KEGG pathway database (Fig. 4). The identified QS signaling networks included the *cepIR*, AI-1, BDSF, and AI-3 systems; however, the AI-2 system was not detected. The KEGG QS pathway showed the *rpfFR*, a significant regulatory gene of the BDSF system that directly affects the c-di-GMP level. In this pathway, *rpfF* is expressed in response to a BDSF signal. Furthermore, a BDSF signal binds *rpfR* and regulates c-di GMP levels (Schmid et al. 2017; Richter et al. 2019).

**Fig. 5:**
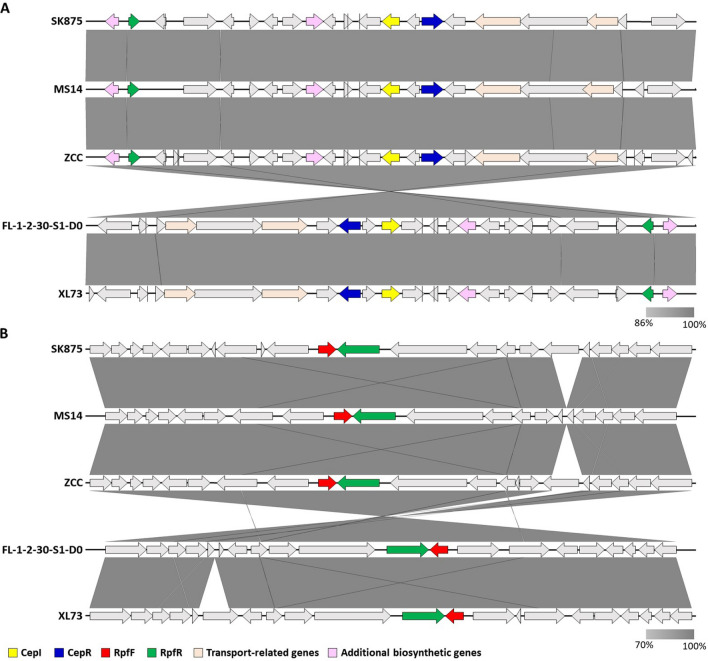
Comparative gene mapping of major QS regulatory system in *B. contaminans* genomes. (**A**) Comparative mapping of the LuxR/LuxI system-related gene. CepI and CepR homologs are shown in yellow and blue colored arrows. Grey lines connect regions with > 86% identity, and darker color indicates a higher percentage of identity. **B** Comparative mapping of the RpfF/RpFR system-related genes. RpfF and RpfR homologs are shown in red and green colored arrows, respectively. Grey lines connect regions with > 70% identity, and darker color indicates a higher percentage of identity

The local topology of QS genes frequently provides continuities that enable researchers to relate genes, proteins, genomes, and characteristics across species and genera using comparative biological analysis (Choudhary et al. 2013; Prescott and Decho 2020). In this study, two quorum-sensing systems were compared by examining the local topology of QS genes in five *B*. *contaminans* strains. Analysis of the map positions of the *cepIR* and *rpfFR* genes, which regulate the AHL and BDSF signaling systems, showed that five *B*. *contaminans* species, including *B*. *contaminans* SK875, were similar regarding their respective gene positions (Fig. 5). The *cepIR* and *rpfFR* genes were located on chromosome 2 in five *B. contaminans* strains. The *cepI* and *cepR* genes are, respectively, indicated by yellow and blue arrows with the adjacent genes (Fig. 5A). The red and green arrows represent the orientation and position of *rpfF* and *rpfR* genes, respectively (Fig. 5B). When comparing the *B*. *contaminans* SK875 and other strains, gene inversion in QS regulatory system was observed in *B*. *contaminans* FL-1-2-30-S1-D0 and XL73. In the genus *Burkholderia*, genomic rearrangements involving inversions or translocations were previously reported (Seo et al. 2015). Rearrangements of bacterial genes are common and have occurred at the strain level as well as species level. Since that gene or genome rearrangements are related to adaptation, it is not surprising that some genes in *B*. *contaminans* exhibit rearrangements (Siqueira et al. 2014). The transcriptional direction of *cepR* gene was opposite to the transcriptional orientation of *cepI* gene on the genome in five *B. contaminans* strains. The *rpfR* gene is also opposite to the transcriptional direction of the *rpfF* gene in five *B. contaminans* strains. The chemical structure of QS signals, the local topology of QS genes, and the location of QS systems within the chromosomes are slightly conserved throughout the genus (Choudhary et al. 2013). The ability to produce AHL signals is widespread in the genus *Burkholderia*. Two QS system genes, including *cepIR* and *rpfFR* in *Burkholderia*, are generally situated on chromosome 2, which includes most genes associated with virulence and secretory systems. This coincides with the result that QS genes regulate pathogenesis and virulence in several species of *Burkholderia* (Whitlock et al. 2007).

**Fig. 6:**
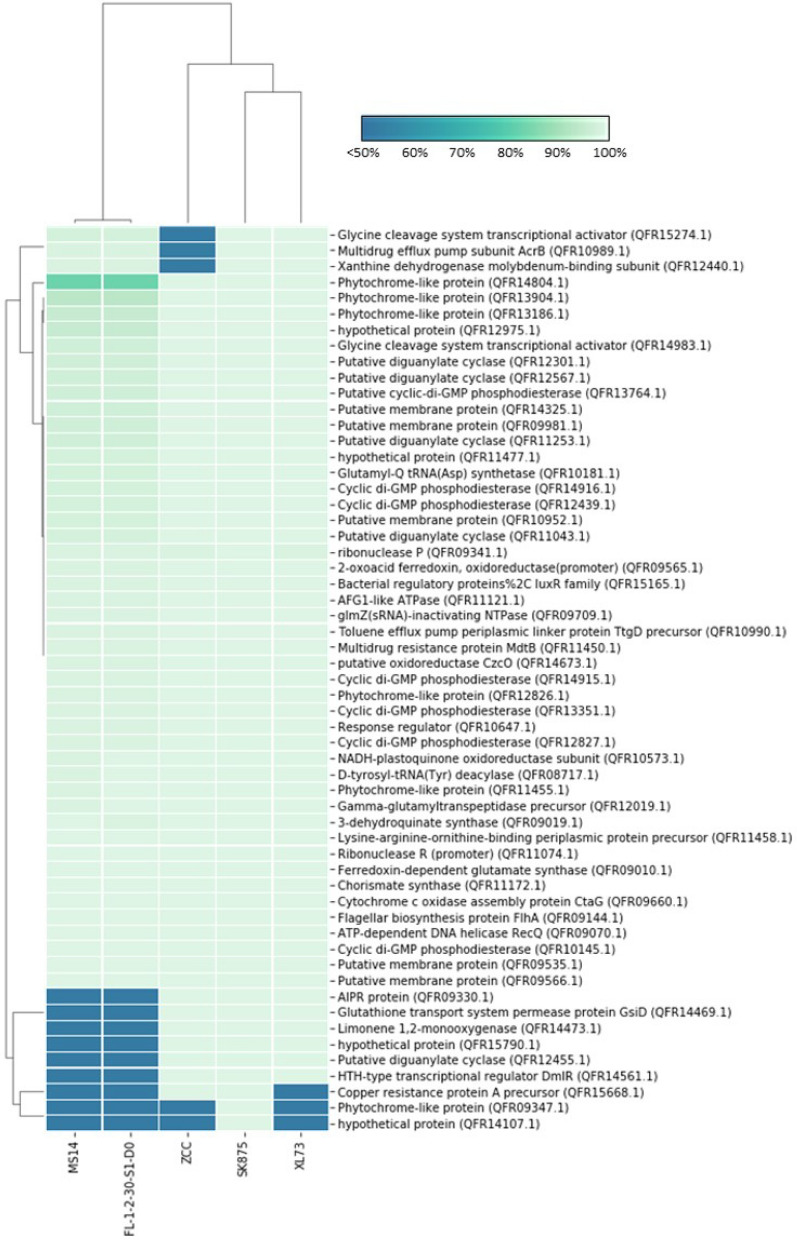
Heatmap of sequence similarity of QS-related proteins between *B. contaminans* SK875 and other *B. contaminans* genomes. The heatmap is visualized with a color bar of light green (high identity) to blue (low identity). The bottom of the heatmap presents the five *B. contaminans* strains. The right of the heatmap presents the QS-related proteins

Previously, we screened and characterized transposon (Tn5) insertion mutants with longer life spans in *C. elegans* (Jung et al. 2017) and investigated decreased autoinducer (AI) secretion in the Tn library of *B. contaminans* SK875 (unpublished). *B. contaminans* SK875 showed 57 quorum sensing-related genes, and 45 of them were also present in other *B. contaminans* genomes (Fig. 6). These genes demonstrated high sequence homology in all strains. Six genes, such as glutathione transport system permease protein GsiD, limonene 1,2-monooxygenase, HTH-type transcriptional regulator DmlR, phytochrome-like protein, and two hypothetical proteins, were present in only *B. contaminans* SK875, ZCC, and XL73 strains. A glycine cleavage system transcriptional activator, multidrug efflux pump subunit AcrB and xanthine dehydrogenase molybdenum-binding subunit showed high sequence homology in all strains except for the *B. contaminans* ZCC strain. Phytochrome-like protein (Accession No. QFR09347.1) and a hypothetical protein (QFR14107.1) were found only in the *B. contaminans* SK875 strain. In conclusion, five *B. contaminans* strains indicted a generally high degree of genetic similarity in QS systems and their related genes.

## Discussion

*Burkholderia* species has increasingly been reported as opportunistic pathogens cause respiratory diseases by following ingestion and inhalation (Mahenthiralingam et al. 2005). *Burkholderia* colonizes rather than immediate respiratory tract infections and causes sepsis in patients with respiratory disease (Mahenthiralingam et al. 2008; Sousa et al. 2011). *Burkholderia* species can produce a wide variety of potential virulence factors, although not all have yet been shown to have a role in the pathogenesis of human disease (Sousa et al. 2011). The genome sequencing is a powerful tool for bacterial taxonomy, antibiotic resistance prediction, and pathogenicity prediction. In this study, we sequenced and analyzed the complete genome sequence of *B*. *contaminans* SK875 strain found in the respiratory tract of a pig with a respiratory disease to understand the pathogen characteristics of *B*. *contaminans*.

By comparing the *B*. *contaminans* SK875 strain and four other strains of *B*. *contaminans* worldwide, we found that SK875 strain was most similar to ZCC strain derived from mining soil sample, which was consistent with the results of phylogeny analysis and ANI analysis. This result suggests that *B*. *contaminans* SK875 could harbor biological characteristics that are similar to those of ZCC strain derived from Chinese environment.

Although the process by which the *B*. *contaminans* genes causes human disease is not well understood, several virulence factors either contributing to growth in adhesion or invasion could be found in the genome of the *B*. *contaminans* SK875 strain, such as *boaA*, *boaB*, Type IV pili, capsules of synthesis-related and flagella-related genes. Also, *B*. *contaminans* SK875 strain had a large number of antibiotic-related genes. Both *boaA* and *boaB* genes are related to adherence function through their role as adhesions in vitro, thus conferring the ability to replicate inside macrophage-like cells (Balder et al. 2010; Campos et al. 2013). Type IV* pili* genes are important for the virulence of many gram-negative bacteria and also play a role in adherence, a vital virulence mechanism resolved by carbohydrate molecules, pilus, and non-pilus adhesins (Carbonnelle et al. 2005). *B. contaminans* SK875, FL-1-2-30-S1-D0, and XL73 harbor all of these genes, but MS14 and ZCC lacked adherence-related genes, such as *boaA* or Type IV *pili*. The lack of these genes in some genomes demonstrates their inability to attach to the host cells to initiate infection (Deng et al. 2016). The capsule and flagella are also considered an important virulence factor in bacteria (Reckseidler et al. 2001). Flagella are correlated with the ability of an organism to cause disease in the motility phenotype imparted by these organelles (Duan et al. 2013). The loss of capsule production was shown to attenuate bacterial infections (Drysdale et al. 2005). Genomes of five *B*. *contaminans* possessed various capsules and flagella genes. This fact indicates that *B*. *contaminans* strains have the strategies to infect mammalian hosts.

*B*. *contaminans* SK875 has a variety of secretion systems that control the expression of virulence factor genes for bacterial pathogenesis (Rutherford and Bassler 2012). The *cepIR* gene of SK875 is able to produce AHL QS molecules, while BsaT3SS system helps to deliver the effector proteins which manipulate host cell functions, thereby evading the immune system and causing diseases into host cells (Kendall 2017). Several studies have identified T6SS as a key virulence determinant that is expressed by a variety of bacterial pathogens, and recently characterized as the mechanism of protein transport from gram-negative bacteria to eukaryotic cells (Ho et al. 2014; Yang et al. 2018). Interestingly, the conserved patterns of QS-related gene neighborhoods in SK875 are apparently at odds with the general view that gene arrangements in prokaryotes are evolutionarily volatile and may change substantially even on short evolutionary scales when gene sequences diverge minimally (Choudhary et al. 2013; Prescott and Decho 2020). From this evolutionary perspective we speculate that the appearance of a novel QS system may cause a major change in the lifestyle of a bacterial species (Choudhary et al. 2013).

In conclusion, genome analysis provided a comprehensive analysis of the disease promising of *B*. *contaminans* SK875. Comparative genomic analysis revealed that the genomes of *B*. *contaminans* were similar regardless of the isolated source, except that *B*. *contaminans* SK875 had some QS-related genes different from the other *B*. *contaminans* genomes. Therefore, our data will be valuable for understanding genetic characteristics, including the QS system in *B*. *contaminans* SK875.

## Supplementary Information

**Additional file 1: Table S1.** Genome features of *Burkholderia *species. **Table S2.** COG distribution of the genes in *B*. *contaminans *SK875. **Table S3.** KEGG distribution of the genes in *B*. *contaminans *SK875. **Table S4.** List of unique genes of *B*. *contaminans *SK875. **Table S5.** COG distribution of the unique genes in *B*. *contaminans *SK875. **Table S6.** Prophage regions of the *B*. *contaminans *genomes. **Table S7.** The CRISPR loci of the five *B*. *contaminans *genomes. **Table S8.** Putative virulence genes in five *B*. *contaminans *genomes predicted by VFDB. **Table S9.** Antibiotic resistance genes in five *B*. *contaminans *genomes.

## Data Availability

The assembled genome has been deposited at the NCBI database under the Accession Number CP028807.1–CP028810.1.
