# Supplementary material for: Comprehensive genome analysis of Burkholderia contaminans SK875, a quorum-sensing strain isolated from the swine
Source: AMB Express. 2023 Mar 11;13:30. doi: 10.1186/s13568-023-01537-8 (PMC10006387; doi:10.1186/s13568-023-01537-8)
Supplement: Supplementary file 1 — Additional file 1: Table S1. Genome features of Burkholderia species. Table S2. COG distribution of the genes in B. contaminans SK875. Table S3. KEGG distribution of the genes in B. contaminans SK875. Table S4. List of unique genes of B. contaminans SK875. Table S5. COG distribution of the unique genes in B. contaminans SK875. Table S6. Prophage regions of the B. contaminans genomes. Table S7. The CRISPR loci of the five B. contaminans genomes. Table S8. Putative virulence genes in five B. contaminans genomes predicted by VFDB. Table S9. Antibiotic resistance genes in five B. contaminans genomes. [file 13568_2023_1537_MOESM1_ESM.pdf]

AMB Express

Comprehensive genome analysis of *Burkholderia contaminans* SK875, a quorum-sensing strain isolated from the swine

Eiseul Kim [a,†], Hae-In Jung [b,†], Si Hong Park [c], Hae-Yeong Kim [a,*], Soo-Ki Kim [b,*]

[a] Institute of Life Sciences & Resources and Department of Food Science and Biotechnology, Kyung Hee University, Yongin 17104, Korea

[b] Department of Animal Sciences and Technology, Konkuk University, Seoul 05029, Korea

[c] Department of Food Science and Technology, Oregon State University, Corvallis, OR 97331, USA

[†]These authors contributed equally to the work

Address correspondence to Hae-Yeong Kim, hykim@khu.ac.kr

Address correspondence to Soo-Ki Kim, sookikim@konkuk.ac.kr

Table S1. Genome features of *Burkholderia* species

#Organism Name	Strain	Size (Mb)	GC%	CDS	Assembly	Accession no.
B. ambifaria	MC40-6	7.64254	66.38	6,575	Complete	CP001025.1-CP001028.1
B. ambifaria	AMMD	7.52857	66.79	6,551	Complete	CP000440.1-CP000443.1
B. ambifaria	AMMD	7.52858	66.79	6,548	Complete	CP009797.1-CP009800.1
B. cenocepacia	J2315	8.05578	66.92	7,199	Complete	AM747720.1-AM747723.1
B. cenocepacia	AU 1054	7.27912	66.92	6,372	Complete	CP000378.1-CP000380.1
B. cenocepacia	MC0-3	7.97139	66.58	7,031	Complete	CP000958.1-CP000960.1
B. cenocepacia	HI2424	7.70284	66.80	6,867	Complete	CP000458.1-CP000461.1
B. cenocepacia	H111	7.71489	67.31	6,805	Complete	HG938370.1-HG938372.1
B. cenocepacia	DDS 22E-1	8.04525	66.97	7,038	Complete	CP007782.1-CP007784.1
B. cenocepacia	DWS 37E-2	6.61242	66.50	5,691	Complete	CP007779.1-CP007781.1
B. cenocepacia	ST32	8.09039	67.01	7,016	Complete	CP011917.1-CP011920.1
B. cenocepacia	842	8.1497	66.98	7,156	Complete	CP015032.1-CP015035.1
B. cenocepacia	895	8.73148	66.74	7,784	Complete	CP015036.1-CP015038.1
B. cenocepacia	FL-5-3-30-S1-D7	6.33075	67.04	5,624	Complete	CP013396.1-CP013397.1
B. cenocepacia	MSMB384WGS	7.7806	67.24	6,895	Complete	CP013450.1-CP013451.1
B. cenocepacia	VC7848	7.49946	66.90	6,631	Complete	CP019668.1
B. cenocepacia	VC12802	7.39491	67.03	6,508	Complete	CP019669.1-CP019670.1
B. cenocepacia	VC12308	7.6345	67.14	6,775	Complete	CP019671.1-CP019674.1
B. cenocepacia	CR318	7.66489	66.82	6,755	Complete	CP017238.1-CP017240.1
B. cenocepacia	PC184 Mulks	7.06705	66.83	6,177	Complete	CP021067.1-CP021069.1
B. cenocepacia	YG-3	8.03646	66.79	7,017	Complete	CP034545.1-CP034547.1
B. cenocepacia	PS27	7.60771	66.96	6,600	Complete	CP060039.1-CP060042.1
B. cenocepacia	K56-2	7.74192	67.02	6,851	Complete	CP053300.1-CP053303.1
B. cepacia	GG4	6.46732	66.71	5,724	Complete	CP003774.1-CP003775.1
B. cepacia	JBK9	8.48121	66.81	7,462	Complete	CP013730.1-CP013732.1
B. cepacia	DDS 7H-2	8.14711	67.06	7,280	Complete	CP007785.1-CP007787.1
B. cepacia	LO6	6.41938	67.00	5,542	Complete	CP011301.1
B. cepacia	UCB 717	8.60595	66.60	7,642	Complete	CP012981.1-CP012984.1
B. cepacia	INT3-BP177	7.33738	66.85	6,401	Complete	CP013375.1-CP013376.1
B. cepacia	MSMB1184WGS	8.00363	66.37	6,826	Complete	CP013442.1-CP013444.1
B. cepacia	FDAARGOS_345	8.56985	66.58	7,655	Complete	CP022081.1-CP022084.2
B. cepacia	FDAARGOS_388	8.56982	66.58	7,615	Complete	CP023519.1-CP023521.1
B. cepacia	ATCC 25416	8.56701	66.58	7,611	Complete	CP007745.1-CP007748.1
B. cepacia	ATCC 25416	8.57439	66.57	7,619	Complete	CP034553.1-CP034557.1
B. cepacia	BC16	8.36687	66.76	7,337	Complete	CP045235.1-CP045237.1
B. cepacia	39628	8.40391	66.79	7,194	Complete	CP032009.1-CP032011.1
B. contaminans	MS14	8.50925	66.40	7,494	Complete	CP009743.1-CP009745.1
B. contaminans	FL-1-2-30-S1-D0	8.17078	66.53	7,138	Complete	CP013390.1-CP013392.1
B. contaminans	ZCC	9.00224	66.06	7,724	Complete	CP042164.1-CP042168.1
B. contaminans	SK875	8.59605	66.30	7,564	Complete	CP028807.1-CP028810.1
B. contaminans	XL73	8.65686	66.27	7,565	Complete	CP046607.1-CP046611.1
B. dolosa	AU0158	6.40909	67.04	5,592	Complete	CP009793.1-CP009795.1
B. dolosa	FDAARGOS_562	6.4091	67.04	5,612	Complete	CP033838.1-CP033840.1
B. lata	383	8.67628	66.26	7,692	Complete	CP000150.1-CP000152.1
B. lata	FL-7-5-30-S1-D0	8.35484	66.50	7,345	Complete	CP013404.1-CP013406.1
B. lata	A05	8.78701	66.20	7,873	Complete	CP024943.1-CP024945.1
B. multivorans	ATCC 17616	7.00881	66.69	6,247	Complete	AP009385.1-AP009388.1
B. multivorans	ATCC 17616	7.00862	66.69	6,273	Complete	CP000868.1-CP000871.1
B. multivorans	DDS 15A-1	7.28187	66.60	6,460	Complete	CP008728.1-CP008730.1
B. multivorans	ATCC BAA-247	6.32275	67.20	5,584	Complete	CP009830.1-CP009832.1
B. multivorans	AU1185	6.62094	66.88	5,828	Complete	CP013430.1-CP013432.1

B. multivorans	MSMB1640WGS	6.84536	66.90	6,008	Complete	CP013466.1-CP013468.1
B. multivorans	FDAARGOS_246	6.32286	67.20	5,828	Complete	CP020397.1-CP020399.1
B. multivorans	FDAARGOS_547	6.39408	67.29	5,592	Complete	CP033748.1-CP033750.1
B. multivorans	FDAARGOS_548	6.65386	66.99	5,837	Complete	CP033745.1-CP033747.1
B. multivorans	FDAARGOS_496	7.79642	67.24	6,732	Complete	CP033854.1-CP033856.1
B. multivorans	FDAARGOS_623	6.47862	67.12	5,918	Complete	CP044053.1-CP044055.1
B. multivorans	FDAARGOS_622	6.47864	67.12	5,916	Complete	CP044056.1-CP044058.1
B. multivorans	FDAARGOS_719	6.56565	67.25	5,785	Complete	CP046336.1-CP046340.1
B. multivorans	FDAARGOS_726	6.51869	67.30	5,737	Complete	CP046341.1-CP046343.1
B. multivorans	FDAARGOS_722	6.59044	67.08	5,873	Complete	CP046344.1-CP046346.1
B. anthina	BJQ0011	6.4565	66.39	5,759	Complete	CP066769.1-CP066770.1
B. anthina	1CH1	8.45737	66.27	7,654	Complete	CP071825.1-CP071829.1
B. pyrrocinia	DSM 10685	7.96135	66.47	6,924	Complete	CP011503.1-CP011506.1
B. pyrrocinia	mHSR5	8.0044	66.33	7,078	Complete	CP024902.1-CP024904.1
B. stabilis	ATCC BAA-67	8.52795	66.41	7,552	Complete	CP016442.1-CP016444.1
B. stabilis	FERMP-21014	7.7211	66.69	6,770	Complete	AP018111.1-AP018113.1
B. ubonensis	MSMB22	7.18907	67.31	6,206	Complete	CP009486.1-CP009488.1
B. ubonensis	RF23-BP41	7.9616	67.08	6,914	Complete	CP013368.1-CP013371.1
B. ubonensis	MSMB2035	7.23506	66.85	6,500	Complete	CP013414.1-CP013416.1
B. ubonensis	MSMB0783	6.68485	67.32	5,631	Complete	CP013420.1-CP013422.1
B. ubonensis	MSMB1189WGS	8.02876	66.79	6,968	Complete	CP013445.1-CP013447.1
B. ubonensis	MSMB1471WGS	7.46341	67.38	6,560	Complete	CP013462.1-CP013465.1
B. vietnamiensis	G4	8.39107	65.73	7,617	Complete	CP000614.1-CP000621.1
B. vietnamiensis	LMG 10929	6.9305	66.84	6,013	Complete	CP009629.1-CP009632.1
B. vietnamiensis	FL-2-3-30-S1-D0	6.81528	67.27	5,846	Complete	CP013393.1-CP013395.1
B. vietnamiensis	AU1233	6.83507	66.86	5,988	Complete	CP013433.1-CP013434.1
B. vietnamiensis	HI2297	6.76453	67.09	5,854	Complete	CP013439.1-CP013441.1
B. vietnamiensis	MSMB608WGS	6.89171	67.02	5,998	Complete	CP013453.1-CP013456.1
B. vietnamiensis	FDAARGOS_239	6.93041	66.84	6,007	Complete	CP020393.1-CP020395.1

Table S2. COG distribution of the genes in *B. contaminans* SK875

Functional class	Category	Ratio (%)
Information storage and processing	J: Translation, ribosomal structure & biogenesis	3.02
	K: Transcription	10.87
	L: Replication, recombination & repair	2.55
Cellular processes and signaling	D: Cell cycle control, cell division, chromosome partitioning	0.54
	M: Cell wall/membrane/envelope biogenesis	5.96
	N: Cell motility	1.63
	O: Post-translational modification, protein turnover & chaperones	2.88
	T: Signal transduction mechanisms	4.30
	U: Intracellular trafficking, secretion & vesicular transport	2.67
	V: Defense mechanisms	0.97
Metabolism	P: Inorganic ion transport & metabolism	6.64
	F: Nucleotide transport & metabolism	1.49
	G: Carbohydrate transport & metabolism	6.45
	E: Amino acid transport & metabolism	11.06
	H: Coenzyme transport & metabolism	2.96
	I: Lipid transport & metabolism	4.78
	C: Energy production & conversion	5.91
	Q: Secondary metabolites biosynthesis, transport & catabolism	3.97
Poorly characterized	R: General function prediction only	14.15
	S: Function unknown	7.20

Table S3. KEGG distribution of the genes in *B. contaminans* SK875

KEGG pathway	Number of genes
Cell cycle [ko04110]	1
Cell cycle - yeast [ko04111]	1
Cell cycle - Caulobacter [ko04112]	9
Meiosis - yeast [ko04113]	1
Oocyte meiosis [ko04114]	1
p53 signaling pathway [ko04115]	3
Apoptosis [ko04210]	1
Bacterial chemotaxis [ko02030]	21
Flagellar assembly [ko02040]	15
Regulation of actin cytoskeleton [ko04810]	2
Adherens junction [ko04520]	1
Tight junction [ko04530]	2
Regulation of autophagy [ko04140]	1
Lysosome [ko04142]	7
Endocytosis [ko04144]	1
Peroxisome [ko04146]	36
ABC transporters [ko02010]	198
Phosphotransferase system (PTS) [ko02060]	5
Bacterial secretion system [ko03070]	18
Two-component system [ko02020]	95
Ras signaling pathway [ko04014]	1
cGMP - PKG signaling pathway [ko04022]	2
cAMP signaling pathway [ko04024]	4
HIF-1 signaling pathway [ko04066]	4
FoxO signaling pathway [ko04068]	5
Phosphatidylinositol signaling system [ko04070]	3
Sphingolipid signaling pathway [ko04071]	1
mTOR signaling pathway [ko04150]	1
PI3K-Akt signaling pathway [ko04151]	1
AMPK signaling pathway [ko04152]	5
Hippo signaling pathway [ko04390]	3
Hippo signaling pathway -fly [ko04391]	1
RNA degradation [ko03018]	14
Protein export [ko03060]	12
Ubiquitin mediated proteolysis [ko04120]	2
Sulfur relay system [ko04122]	8
Protein processing in endoplasmic reticulum [ko04141]	1
DNA replication [ko03030]	13
Base excision repair [ko03410]	10
Nucleotide excision repair [ko03420]	9
Mismatch repair [ko03430]	14
Homologous recombination [ko03440]	17
Fanconi anemia pathway [ko03460]	1
RNA polymerase [ko03020]	3
Basal transcription factors [ko03022]	3
Aminoacyl-tRNA biosynthesis [ko00970]	34

Ribosome biogenesis in eukaryotes [ko03008]	2
Ribosome [ko03010]	50
RNA transport [ko03013]	1
Transcriptional misregulation in cancers [ko05202]	1
Viral carcinogenesis [ko05203]	5
Chemical carcinogenesis [ko05204]	15
Proteoglycans in cancer [ko05205]	3
MicroRNAs in cancer [ko05206]	2
Central carbon metabolism in cancer [ko05230]	7
Choline metabolism in cancer [ko05231]	2
Viral myocarditis [ko05416]	1
beta-Lactam resistance [ko01501]	16
Vancomycin resistance [ko01502]	6
Cationic antimicrobial peptide (CAMP) resistance [ko01503]	14
Type II diabetes mellitus [ko04930]	1
Non-alcoholic fatty liver disease (NAFLD) [ko04932]	16
Type I diabetes mellitus [ko04940]	1
Primary immunodeficiency [ko05340]	2
Bacterial invasion of epithelial cells [ko05100]	1
Vibrio cholerae pathogenic cycle [ko05111]	3
Pathogenic Escherichia coli infection [ko05130]	2
Shigellosis [ko05131]	1
Salmonella infection [ko05132]	1
Pertussis [ko05133]	1
Legionellosis [ko05134]	3
Staphylococcus aureus infection [ko05150]	1
Tuberculosis [ko05152]	4
Herpes simplex infection [ko05168]	2
Alzheimer's disease [ko05010]	21
Parkinson's disease [ko05012]	20
Amyotrophic lateral sclerosis (ALS) [ko05014]	3
Huntington's disease [ko05016]	22
Cocaine addiction [ko05030]	1
Amphetamine addiction [ko05031]	1
Alcoholism [ko05034]	5
Alanine, aspartate and glutamate metabolism [ko00250]	38
Glycine, serine and threonine metabolism [ko00260]	44
Cysteine and methionine metabolism [ko00270]	30
Valine, leucine and isoleucine degradation [ko00280]	60
Valine, leucine and isoleucine biosynthesis [ko00290]	24
Lysine biosynthesis [ko00300]	24
Lysine degradation [ko00310]	31
Arginine and proline metabolism [ko00330]	51
Histidine metabolism [ko00340]	30
Tyrosine metabolism [ko00350]	24
Phenylalanine metabolism [ko00360]	26
Tryptophan metabolism [ko00380]	35
Phenylalanine, tyrosine and tryptophan biosynthesis [ko00400]	21
Caffeine metabolism [ko00232]	3

Monobactam biosynthesis [ko00261]	12
Carbapenem biosynthesis [ko00332]	2
Novobiocin biosynthesis [ko00401]	3
Streptomycin biosynthesis [ko00521]	9
Phenylpropanoid biosynthesis [ko00940]	3
Isoquinoline alkaloid biosynthesis [ko00950]	4
Tropane, piperidine and pyridine alkaloid biosynthesis [ko00960]	5
Glycolysis / Gluconeogenesis [ko00010]	48
Citrate cycle (TCA cycle) [ko00020]	25
Pentose phosphate pathway [ko00030]	27
Pentose and glucuronate interconversions [ko00040]	31
Fructose and mannose metabolism [ko00051]	18
Galactose metabolism [ko00052]	13
Ascorbate and aldarate metabolism [ko00053]	20
Starch and sucrose metabolism [ko00500]	17
Amino sugar and nucleotide sugar metabolism [ko00520]	25
Inositol phosphate metabolism [ko00562]	8
Pyruvate metabolism [ko00620]	47
Glyoxylate and dicarboxylate metabolism [ko00630]	46
Propanoate metabolism [ko00640]	27
Butanoate metabolism [ko00650]	47
C5-Branched dibasic acid metabolism [ko00660]	10
Oxidative phosphorylation [ko00190]	45
Photosynthesis [ko00195]	3
Methane metabolism [ko00680]	22
Carbon fixation in photosynthetic organisms [ko00710]	15
Carbon fixation pathways in prokaryotes [ko00720]	26
Nitrogen metabolism [ko00910]	11
Sulfur metabolism [ko00920]	52
N-Glycan biosynthesis [ko00510]	3
Other glycan degradation [ko00511]	3
Various types of N-glycan biosynthesis [ko00513]	1
Other types of O-glycan biosynthesis [ko00514]	4
Glycosaminoglycan degradation [ko00531]	1
Lipopolysaccharide biosynthesis [ko00540]	12
Peptidoglycan biosynthesis [ko00550]	26
Glycosphingolipid biosynthesis - globo series [ko00603]	1
Glycosphingolipid biosynthesis - ganglio series [ko00604]	2
Fatty acid biosynthesis [ko00061]	39
Fatty acid elongation [ko00062]	4
Fatty acid degradation [ko00071]	52
Synthesis and degradation of ketone bodies [ko00072]	15
Steroid biosynthesis [ko00100]	2
Primary bile acid biosynthesis [ko00120]	3
Steroid hormone biosynthesis [ko00140]	6
Glycerolipid metabolism [ko00561]	20
Glycerophospholipid metabolism [ko00564]	13
Ether lipid metabolism [ko00565]	1
Arachidonic acid metabolism [ko00590]	10

Linoleic acid metabolism [ko00591]	1
alpha-Linolenic acid metabolism [ko00592]	2
Sphingolipid metabolism [ko00600]	3
Biosynthesis of unsaturated fatty acids [ko01040]	13
Ubiquinone and other terpenoid-quinone biosynthesis [ko00130]	15
One carbon pool by folate [ko00670]	18
Thiamine metabolism [ko00730]	10
Riboflavin metabolism [ko00740]	7
Vitamin B6 metabolism [ko00750]	10
Nicotinate and nicotinamide metabolism [ko00760]	19
Pantothenate and CoA biosynthesis [ko00770]	23
Biotin metabolism [ko00780]	24
Lipoic acid metabolism [ko00785]	2
Folate biosynthesis [ko00790]	13
Retinol metabolism [ko00830]	15
Porphyrin and chlorophyll metabolism [ko00860]	18
beta-Alanine metabolism [ko00410]	34
Taurine and hypotaurine metabolism [ko00430]	7
Phosphonate and phosphinate metabolism [ko00440]	3
Selenocompound metabolism [ko00450]	15
Cyanoamino acid metabolism [ko00460]	7
D-Glutamine and D-glutamate metabolism [ko00471]	4
D-Alanine metabolism [ko00473]	1
Glutathione metabolism [ko00480]	20
Tetracycline biosynthesis [ko00253]	2
Geraniol degradation [ko00281]	16
Polyketide sugar unit biosynthesis [ko00523]	6
Terpenoid backbone biosynthesis [ko00900]	15
Limonene and pinene degradation [ko00903]	12
Zeatin biosynthesis [ko00908]	1
Sesquiterpenoid and triterpenoid biosynthesis [ko00909]	1
Insect hormone biosynthesis [ko00981]	2
Biosynthesis of ansamycins [ko01051]	2
Biosynthesis of siderophore group nonribosomal peptides [ko01053]	4
Nonribosomal peptide structures [ko01054]	9
Biosynthesis of vancomycin group antibiotics [ko01055]	1
Purine metabolism [ko00230]	61
Pyrimidine metabolism [ko00240]	47
Carbon metabolism [ko01200]	111
2-Oxocarboxylic acid metabolism [ko01210]	40
Fatty acid metabolism [ko01212]	59
Degradation of aromatic compounds [ko01220]	15
Biosynthesis of amino acids [ko01230]	131
Chlorocyclohexane and chlorobenzene degradation [ko00361]	2
Benzoate degradation [ko00362]	20
Bisphenol degradation [ko00363]	1
Fluorobenzoate degradation [ko00364]	2
Dioxin degradation [ko00621]	5
Xylene degradation [ko00622]	5

Toluene degradation [ko00623]	2
Chloroalkane and chloroalkene degradation [ko00625]	24
Naphthalene degradation [ko00626]	7
Aminobenzoate degradation [ko00627]	8
Nitrotoluene degradation [ko00633]	4
Ethylbenzene degradation [ko00642]	2
Styrene degradation [ko00643]	7
Caprolactam degradation [ko00930]	4
Metabolism of xenobiotics by cytochrome P450 [ko00980]	16
Drug metabolism - cytochrome P450 [ko00982]	16
Drug metabolism - other enzymes [ko00983]	8
Cardiac muscle contraction [ko04260]	9
Adrenergic signaling in cardiomyocytes [ko04261]	2
Vascular smooth muscle contraction [ko04270]	2
Salivary secretion [ko04970]	3
Gastric acid secretion [ko04971]	1
Pancreatic secretion [ko04972]	3
Carbohydrate digestion and absorption [ko04973]	1
Protein digestion and absorption [ko04974]	1
Fat digestion and absorption [ko04975]	2
Bile secretion [ko04976]	5
Vitamin digestion and absorption [ko04977]	1
Mineral absorption [ko04978]	4
PPAR signaling pathway [ko03320]	20
Insulin signaling pathway [ko04910]	8
Insulin secretion [ko04911]	2
GnRH signaling pathway [ko04912]	1
Ovarian Steroidogenesis [ko04913]	1
Prolactin signaling pathway [ko04917]	1
Thyroid hormone synthesis [ko04918]	2
Thyroid hormone signaling pathway [ko04919]	1
Adipocytokine signaling pathway [ko04920]	11
Glucagon signaling pathway [ko04922]	5
Plant-pathogen interaction [ko04626]	2
Circadian rhythm [ko04710]	1
Proximal tubule bicarbonate reclamation [ko04964]	2
Chemokine signaling pathway [ko04062]	1
Fc gamma R-mediated phagocytosis [ko04666]	3
Retrograde endocannabinoid signaling [ko04723]	2
Glutamatergic synapse [ko04724]	3
GABAergic synapse [ko04727]	5
Dopaminergic synapse [ko04728]	1

Table S4. List of unique genes of *B. contaminans* SK875

Proteins	Accession no.
hypothetical protein SK875_A00077	QFR08313.1
hypothetical protein SK875_A00158	QFR08392.1
hypothetical protein SK875_A00159	QFR08393.1
hypothetical protein SK875_A00161	QFR08395.1
hypothetical protein SK875_A00201	QFR08430.1
hypothetical protein SK875_A00210	QFR08439.1
hypothetical protein SK875_A00336	QFR08563.1
hypothetical protein SK875_A00392	QFR08617.1
hypothetical protein SK875_A00407	QFR08632.1
hypothetical protein SK875_A00426	QFR08651.1
hypothetical protein SK875_A00449	QFR08674.1
hypothetical protein SK875_A00451	QFR08676.1
hypothetical protein SK875_A00452	QFR08677.1
hypothetical protein SK875_A00612	QFR08836.1
hypothetical protein SK875_A00670	QFR08889.1
hypothetical protein SK875_A00671	QFR08890.1
hypothetical protein SK875_A00735	QFR08953.1
hypothetical protein SK875_A00868	QFR09081.1
Toxin HigB-2	QFR09185.1
hypothetical protein SK875_A01146	QFR09347.1
hypothetical protein SK875_A01154	QFR09355.1
hypothetical protein SK875_A01208	QFR09407.1
hypothetical protein SK875_A01365	QFR09564.1
hypothetical protein SK875_A01611	QFR09809.1
hypothetical protein SK875_A01843	QFR10040.1
hypothetical protein SK875_A01877	QFR10073.1
hypothetical protein SK875_A02034	QFR10228.1
hypothetical protein SK875_A02257	QFR10441.1
hypothetical protein SK875_A02261	QFR10445.1
hypothetical protein SK875_A02264	QFR10448.1
hypothetical protein SK875_A02274	QFR10458.1
hypothetical protein SK875_A02286	QFR10470.1
hypothetical protein SK875_A02640	QFR10814.1
hypothetical protein SK875_A02718	QFR10892.1
hypothetical protein SK875_A02754	QFR10928.1
hypothetical protein SK875_A02784	QFR10958.1
hypothetical protein SK875_A02809	QFR10983.1
hypothetical protein SK875_A02838	QFR11012.1
hypothetical protein SK875_A02904	QFR11075.1
hypothetical protein SK875_A02941	QFR11110.1
hypothetical protein SK875_A02982	QFR11150.1
hypothetical protein SK875_A03014	QFR11181.1
hypothetical protein SK875_A03133	QFR11300.1
hypothetical protein SK875_A03138	QFR11304.1
hypothetical protein SK875_A03142	QFR11308.1
hypothetical protein SK875_A03268	QFR11433.1

hypothetical protein SK875_A03280	QFR11445.1
hypothetical protein SK875_A03323	QFR11488.1
hypothetical protein SK875_B00004	QFR11492.1
50S ribosomal protein L25	QFR11493.1
hypothetical protein SK875_B00006	QFR11494.1
50S ribosomal protein L25	QFR11495.1
hypothetical protein SK875_B00008	QFR11496.1
hypothetical protein SK875_B00009	QFR11497.1
hypothetical protein SK875_B00010	QFR11498.1
hypothetical protein SK875_B00011	QFR11499.1
KDP operon transcriptional regulatory protein KdpE	QFR11517.1
hypothetical protein SK875_B00037	QFR11525.1
hypothetical protein SK875_B00122	QFR11610.1
hypothetical protein SK875_B00211	QFR11699.1
hypothetical protein SK875_B00238	QFR11726.1
hypothetical protein SK875_B00409	QFR11894.1
hypothetical protein SK875_B00440	QFR11925.1
hypothetical protein SK875_B00450	QFR11935.1
hypothetical protein SK875_B00491	QFR11975.1
hypothetical protein SK875_B00515	QFR11999.1
Hca operon transcriptional activator	QFR12024.1
hypothetical protein SK875_B00637	QFR12121.1
hypothetical protein SK875_B00671	QFR12155.1
hypothetical protein SK875_B00704	QFR12188.1
hypothetical protein SK875_B00728	QFR12212.1
Sulfite exporter TauE/SafE	QFR12306.1
hypothetical protein SK875_B00967	QFR12450.1
hypothetical protein SK875_B00976	QFR12459.1
Serine/threonine-protein kinase HipA	QFR12476.1
hypothetical protein SK875_B01007	QFR12490.1
hypothetical protein SK875_B01199	QFR12682.1
hypothetical protein SK875_B01319	QFR12802.1
hypothetical protein SK875_B01334	QFR12817.1
hypothetical protein SK875_B01335	QFR12818.1
hypothetical protein SK875_B01364	QFR12847.1
hypothetical protein SK875_B01398	QFR12881.1
hypothetical protein SK875_B01422	QFR12904.1
hypothetical protein SK875_B01556	QFR13037.1
hypothetical protein SK875_B01683	QFR13164.1
hypothetical protein SK875_B01694	QFR13175.1
IgA FC receptor precursor	QFR13237.1
hypothetical protein SK875_B01773	QFR13253.1
hypothetical protein SK875_B01866	QFR13346.1
hypothetical protein SK875_B01949	QFR13428.1
hypothetical protein SK875_B01955	QFR13434.1
hypothetical protein SK875_B01976	QFR13455.1
hypothetical protein SK875_B02102	QFR13581.1
Oxygen-dependent choline dehydrogenase	QFR13624.1
hypothetical protein SK875_B02161	QFR13640.1

hypothetical protein SK875_B02162	QFR13641.1
hypothetical protein SK875_B02241	QFR13720.1
hypothetical protein SK875_B02256	QFR13735.1
hypothetical protein SK875_B02300	QFR13779.1
hypothetical protein SK875_B02313	QFR13792.1
Long-chain-fatty-acid--CoA ligase	QFR13825.1
hypothetical protein SK875_B02347	QFR13826.1
Serine/threonine-protein kinase PknB	QFR13857.1
hypothetical protein SK875_B02402	QFR13881.1
hypothetical protein SK875_B02504	QFR13983.1
hypothetical protein SK875_B02558	QFR14037.1
hypothetical protein SK875_B02573	QFR14052.1
hypothetical protein SK875_B02629	QFR14107.1
hypothetical protein SK875_B02697	QFR14175.1
hypothetical protein SK875_B02765	QFR14243.1
putative cobalt transporter subunit (CbtA)	QFR14263.1
hypothetical protein SK875_C00047	QFR14376.1
hypothetical protein SK875_C00052	QFR14381.1
hypothetical protein SK875_C00086	QFR14415.1
hypothetical protein SK875_C00170	QFR14499.1
hypothetical protein SK875_C00213	QFR14542.1
hypothetical protein SK875_C00217	QFR14546.1
hypothetical protein SK875_C00218	QFR14547.1
hypothetical protein SK875_C00242	QFR14571.1
hypothetical protein SK875_C00314	QFR14643.1
hypothetical protein SK875_C00324	QFR14653.1
hypothetical protein SK875_C00338	QFR14667.1
hypothetical protein SK875_C00354	QFR14683.1
hypothetical protein SK875_C00356	QFR14685.1
hypothetical protein SK875_C00357	QFR14686.1
hypothetical protein SK875_C00358	QFR14687.1
hypothetical protein SK875_C00410	QFR14738.1
hypothetical protein SK875_C00439	QFR14767.1
hypothetical protein SK875_C00444	QFR14772.1
hypothetical protein SK875_C00474	QFR14802.1
hypothetical protein SK875_C00475	QFR14803.1
hypothetical protein SK875_C00506	QFR14834.1
hypothetical protein SK875_C00516	QFR14844.1
hypothetical protein SK875_C00517	QFR14845.1
hypothetical protein SK875_C00578	QFR14906.1
hypothetical protein SK875_C00610	QFR14938.1
hypothetical protein SK875_C00666	QFR14994.1
3-deoxy-D-manno-octulosonate 8-phosphate phosphatase KdsC	QFR15050.1
hypothetical protein SK875_C00821	QFR15149.1
N-acetyldiaminopimelate deacetylase	QFR15209.1
hypothetical protein SK875_C00949	QFR15276.1
hypothetical protein SK875_C00956	QFR15283.1
hypothetical protein SK875_C00966	QFR15293.1
hypothetical protein SK875_C01011	QFR15338.1

hypothetical protein SK875_C01024	QFR15351.1
hypothetical protein SK875_C01025	QFR15352.1
Putative cyclase	QFR15355.1
hypothetical protein SK875_C01035	QFR15362.1
hypothetical protein SK875_C01043	QFR15370.1
hypothetical protein SK875_C01081	QFR15408.1
hypothetical protein SK875_C01082	QFR15409.1
Integrase core domain protein	QFR15434.1
short chain dehydrogenase	QFR15557.1
hypothetical protein SK875_C01288	QFR15608.1
hypothetical protein SK875_C01293	QFR15613.1
hypothetical protein SK875_p00004 (plasmid)	QFR15644.1
hypothetical protein SK875_p00007 (plasmid)	QFR15647.1
hypothetical protein SK875_p00018 (plasmid)	QFR15658.1
hypothetical protein SK875_p00019 (plasmid)	QFR15659.1
hypothetical protein SK875_p00020 (plasmid)	QFR15660.1
hypothetical protein SK875_p00033 (plasmid)	QFR15673.1
hypothetical protein SK875_p00035 (plasmid)	QFR15675.1
hypothetical protein SK875_p00041 (plasmid)	QFR15681.1
hypothetical protein SK875_p00044 (plasmid)	QFR15684.1
hypothetical protein SK875_p00056 (plasmid)	QFR15696.1
hypothetical protein SK875_p00063 (plasmid)	QFR15703.1
hypothetical protein SK875_p00065 (plasmid)	QFR15705.1
hypothetical protein SK875_p00067 (plasmid)	QFR15707.1
hypothetical protein SK875_p00089 (plasmid)	QFR15729.1
hypothetical protein SK875_p00099 (plasmid)	QFR15739.1
Error-prone DNA polymerase (plasmid)	QFR15767.1
hypothetical protein SK875_p00145 (plasmid)	QFR15785.1
hypothetical protein SK875_p00153 (plasmid)	QFR15793.1
hypothetical protein SK875_p00157 (plasmid)	QFR15797.1
hypothetical protein SK875_p00168 (plasmid)	QFR15807.1
hypothetical protein SK875_p00171 (plasmid)	QFR15810.1
IS2 repressor TnpA (plasmid)	QFR15818.1
Thiol:disulfide interchange protein DsbD precursor (plasmid)	QFR15821.1
hypothetical protein SK875_p00193 (plasmid)	QFR15832.1
hypothetical protein SK875_p00195 (plasmid)	QFR15834.1
hypothetical protein SK875_p00196 (plasmid)	QFR15835.1
hypothetical protein SK875_p00216 (plasmid)	QFR15855.1
Integrase core domain protein (plasmid)	QFR15857.1
RNA polymerase sigma factor (plasmid)	QFR15862.1
hypothetical protein SK875_p00227 (plasmid)	QFR15866.1
hypothetical protein SK875_p00228 (plasmid)	QFR15867.1

Table S5. COG distribution of the unique genes in *B. contaminans* SK875

Functional class	Category	Ratio (%)
Information storage and processing	J: Translation, ribosomal structure & biogenesis	2.22
	K: Transcription	6.65
	L: Replication, recombination & repair	17.74
Cellular processes and signaling	D: Cell cycle control, cell division, chromosome partitioning	4.43
	M: Cell wall/membrane/envelope biogenesis	0.00
	N: Cell motility	0.00
	O: Post-translational modification, protein turnover & chaperones	2.22
	T: Signal transduction mechanisms	2.22
	U: Intracellular trafficking, secretion & vesicular transport	0.00
	V: Defense mechanisms	0.00
Metabolism	P: Inorganic ion transport & metabolism	4.43
	F: Nucleotide transport & metabolism	0.00
	G: Carbohydrate transport & metabolism	4.43
	E: Amino acid transport & metabolism	6.65
	H: Coenzyme transport & metabolism	0.00
	I: Lipid transport & metabolism	4.43
	C: Energy production & conversion	6.65
	Q: Secondary metabolites biosynthesis, transport & catabolism	6.65
Poorly characterized	R: General function prediction only	22.17
	S: Function unknown	8.87

Table S6. Prophage regions of the *B. contaminans* genomes

Strains	Location	Completeness	Start (bp)	End (bp)	GC %	Most Common Phage
MS14	Chromosome 1	incomplete	2,451,943	2,471,894	59.71	Burkho_AP3 (NC_047752(1))
	Chromosome 2	intact	3,157,388	3,187,151	61.42	Salmon_118970_sal3 (NC_031940(13))
	Chromosome 3	incomplete	953,153	960,936	67.74	Burkho_phi1026b (NC_005284(2))
FL-1-2-30-S1-D0	Chromosome 1	questionable	2,105,046	2,149,176	60.58	Escher_vB_EcoM_ECOO78 (NC_041926(9))
	Chromosome 2	incomplete	2,884,643	2,895,876	63.97	Acinet_vB_AbaM_ME3 (NC_041884(3))
	Chromosome 3	incomplete	450,919	458,702	67.74	Burkho_phi1026b (NC_005284(2))
ZCC	Chromosome 1	incomplete	3,655,141	3,668,410	61.76	Gordon_Bowser (NC_030930(2))
	Chromosome 1	incomplete	3,712,981	3,721,713	61.98	Bacill_SP_15 (NC_031245(1))
	Plasmid 2	incomplete	45,760	53,972	59.32	Burkho_BcepIL02 (NC_012743(2))
SK875	Chromosome 1	incomplete	270,930	279,358	61.69	Entero_phi92 (NC_023693(4))
	Chromosome 1	incomplete	2,586,105	2,592,869	66.03	Ralsto_RP12 (NC_041911(2))
XL73	Chromosome 3	incomplete	2,410,143	2,418,703	62.41	Entero_fiAA91_ss (NC_022750(2))
	Plasmid 1	questionable	66,247	88,331	62.81	Stx2_c_Stx2a_F451 (NC_049924(3))
	Plasmid 2	incomplete	58,918	67,148	59.41	Pseudo_JG004 (NC_019450(1))

Table S7. The CRISPR loci of the five *B. contaminans* genomes

Strain	Location	Start	End	DR No.	Repeat consensus/Cas genes	DR length (bp)
MS14	Chromosome 1	27,879	27,977	1	GCTGCACCGCACGTCAATCTGGC	98
FL-1-2-30-S1-D0	Chromosome 2	2,802,389	2,802,487	1	GCCAGATTGACGTGCGGTGCAGC	98
FL-1-2-30-S1-D0	Chromosome 2	3,166,134	3,166,234	1	CGCCGGCCGTGCCGGGGCTGCCG	100
ZCC	Chromosome 1	932,298	932,390	1	CACTCGGCAACCTCGGCGGCTCGA	92
SK875	Chromosome 2	2,456,771	2,457,221	5	AGCAATGTCGGCGGCCTCGTCGGCA	450
SK875	Chromosome 2	2,981,872	2,982,191	4	ATCGGCGGCGCCATCGGCGGCGGCGC	319
XL73	Chromosome 2	302,358	302,551	2	GCCGACGAGGCCGCCGACCAGGCTG	193
XL73	Chromosome 2	302,622	302,877	3	GCCGACGAGGCCGCCGACCAGGCTG	255
XL73	Chromosome 2	305,476	305,839	4	TGCCGACGAGGCCGCCGACATAGCT	363
XL73	Chromosome 2	3,029,591	3,029,796	3	GCGCCGCCGCCGAGGGCGCCGCCGAT	205

Table S8. Putative virulence genes in five *B. contaminans* genomes predicted by VFDB

Factors	Related genes	SK875	MS14	FL-1-2-30-S1-D0	ZCC	XL73
BoaA	boaA	SK875_C00706	NL30_34935, NL30_35080	WI95_32125, WI95_32270	ND	GON15_05295
BoaB	boaB	SK875_B02235, SK875_p00121	NL30_15910	WI95_15970, WI95_17360, WI95_28155	FPQ37_40715	GON15_37945
Type IV pili	pilA	SK875_A01681	NL30_26290	WI95_13650	ND	GON15_35135
	pilB	SK875_A00593	NL30_21195	WI95_02555	FPQ37_02915	GON15_23885
	pilC	SK875_A00594	ND	WI95_02550	FPQ37_02910	GON15_23880
	pilD	SK875_A00595	NL30_21205	WI95_02545	FPQ37_02905	GON15_23875
Capsule I	Undetermined	SK875_A00382	ND	ND	FPQ37_04010	GON15_24975
	Undetermined	SK875_A00276, SK875_A00390, SK875_B01430	NL30_07355, NL30_11345	WI95_04035, WI95_20950	FPQ37_23705, FPQ37_03970, FPQ37_04560	GON15_24915, GON15_25510, GON15_11735
	gmhA	ND	ND	ND	ND	ND
	manC	ND	NL30_08080 NL30_21540	WI95_20230	ND	ND
	wcbA	SK875_A00404	ND	ND	FPQ37_03905	GON15_24850
	wcbB	SK875_A00403	ND	ND	FPQ37_03910	GON15_24855
	wcbC	SK875_A00402	ND	ND	FPQ37_03915	GON15_24860
	wcbD	SK875_A00401	ND	ND	FPQ37_03920	GON15_24865
	wcbO	SK875_A00388	ND	ND	FPQ37_03980	GON15_24925
	wcbP	SK875_A00387	ND	ND	FPQ37_03985	GON15_24950
	wcbQ	SK875_A00386	ND	ND	FPQ37_03990	GON15_24955
	wcbR	SK875_A00385, SK875_C00826	NL30_34420, NL30_35045, NL30_36200, NL30_36225	WI95_32160, WI95_32860	FPQ37_39430, FPQ37_03995	GON15_24960, GON15_05910
	wcbS	SK875_A00384	ND	ND	FPQ37_04000	GON15_24965
	wcbT	SK875_A00383	ND	ND	FPQ37_04005	GON15_24970
	wzm	SK875_A00400	ND	ND	FPQ37_03925	GON15_24870
	wzt2	SK875_A00399	ND	ND	FPQ37_03930	GON15_24875
Flagella	cheA	SK875_A00951	NL30_18600	WI95_00915	FPQ37_01020	GON15_22060

cheB	SK875_A00946	NL30_18575	WI95_00940	FPQ37_01045	GON15_22085
cheD	SK875_A00947	NL30_18580	WI95_00935	FPQ37_01040	GON15_22080
cheR	SK875_A00948	NL30_18585	WI95_00930	FPQ37_01035	GON15_22075
cheW	SK875_A00950	NL30_18595	WI95_00920	FPQ37_01025	GON15_22065
cheY1	SK875_A00952	NL30_18605	WI95_00910	FPQ37_01015	GON15_22055
cheY	SK875_A00945	NL30_18570	WI95_00945	FPQ37_01050	GON15_22090
cheZ	SK875_A00944	NL30_18565	WI95_00950	FPQ37_01055	GON15_22095
flgA	SK875_A01286	NL30_28210	WI95_15570	FPQ37_16635	GON15_37155
flgB	SK875_A01287	NL30_28205	WI95_15565	FPQ37_16630	GON15_37150
flgC	SK875_A01288	NL30_28200	WI95_15560	FPQ37_16625	GON15_37145
flgD	SK875_A01289	NL30_28195	WI95_15555	FPQ37_16620	GON15_37140
flgE	SK875_A01290, SK875_B01443	NL30_07420, NL30_28190	WI95_15550, WI95_20885	FPQ37_23770, FPQ37_16615	GON15_37135, GON15_11670
flgF	SK875_A01291	NL30_28185	WI95_15545	FPQ37_16610	GON15_37130
flgG	SK875_A01292	NL30_28180	WI95_15540	FPQ37_16605	GON15_37125
flgH	SK875_A01293	NL30_28175	WI95_15535	FPQ37_16600	GON15_37120
flgI	SK875_A01294	NL30_28170	WI95_15530	FPQ37_16595	GON15_37115
flgJ	SK875_A01295	NL30_28165	WI95_15525	FPQ37_16590	GON15_37110
flgK	SK875_A01297	NL30_28155	WI95_15515	FPQ37_16580	GON15_37100
flgL	SK875_A01298	NL30_28150	WI95_15510	FPQ37_16575	GON15_37095
flgM	SK875_A01285	NL30_28215	WI95_15575	FPQ37_16640	GON15_37160
flgN	SK875_A01284	NL30_28220	WI95_15580	FPQ37_16645	GON15_37165
flhA	SK875_A00937	NL30_18530	WI95_00985	FPQ37_01090	GON15_22130
flhB	SK875_A00938	NL30_18535	WI95_00980	FPQ37_01085	GON15_22125
flhF	SK875_A00936	NL30_18525	WI95_00990	FPQ37_01095	GON15_22135
flhG	SK875_A00935	NL30_18520	WI95_00995	FPQ37_01100	GON15_22140
fliA	SK875_A00934	NL30_18515	WI95_01000	FPQ37_01105	GON15_22145
fliC	ND	NL30_18665	ND	ND	ND
fliD	SK875_A00965, SK875_C00910	NL30_34040, NL30_18670	WI95_00845, WI95_33285	FPQ37_39870, FPQ37_00945	GON15_21990, GON15_06340
fliE	SK875_A01248	NL30_28410	WI95_15765	FPQ37_16835	GON15_37345
fliF	SK875_A01247	NL30_28415	WI95_15770	FPQ37_16845	GON15_37350
fliG	SK875_A01246	NL30_28420	WI95_15775	FPQ37_16850	GON15_37355
fliH	SK875_A01245	NL30_28425	WI95_15780	FPQ37_16855	GON15_37360
fliI	SK875_A01244	NL30_28430	WI95_15785	FPQ37_16860	GON15_37365

	fliJ	SK875_A01243	NL30_28435	WI95_15790	FPQ37_16865	GON15_37370
	fliK	SK875_A01242	NL30_28440	WI95_15795	FPQ37_16870	GON15_37375
	fliL	SK875_A01091	NL30_19290	WI95_00255	FPQ37_00275	GON15_21350
	fliM	SK875_A01092	NL30_19295	WI95_00250	FPQ37_00270	GON15_21345
	fliN	SK875_A01093	NL30_19300	WI95_00245	FPQ37_00265	GON15_21340
	fliO	SK875_A01094	NL30_19305	WI95_00240	FPQ37_00260	GON15_21335
	fliP	SK875_A01095	NL30_19310	WI95_00235	FPQ37_00255	GON15_21330
	fliQ	SK875_A01096	NL30_19315	WI95_00230	FPQ37_00250	GON15_21325
	fliR	SK875_A01097	NL30_19320	WI95_00225	FPQ37_00245	GON15_21320
	fliS	SK875_A01249	NL30_28405	WI95_15760	FPQ37_16830	GON15_37340
	motA	SK875_A00954, SK875_B02110	NL30_18615, NL30_23870	WI95_00900, WI95_17885	FPQ37_27215, FPQ37_01005	GON15_22045, GON15_08320
	motB	SK875_A00953	NL30_18610	WI95_00905	FPQ37_01010	GON15_22050
	tsr	SK875_A00194, SK875_A00949, SK875_A02839, SK875_B00449, SK875_B00666, SK875_B00938, SK875_C00037, SK875_C00067, SK875_C00461, SK875_C00818	NL30_32435, NL30_32595, NL30_34460 NL30_02170, NL30_03040, NL30_04420, NL30_10920, NL30_15080 NL30_18590, NL30_23055	WI95_00925, WI95_04460, WI95_07700, WI95_32820, WI95_34725, WI95_34785, WI95_34870, WI95_18720, WI95_23835, WI95_25215, WI95_26120	FPQ37_35400, FPQ37_35550, FPQ37_37565, FPQ37_39390, FPQ37_19755, FPQ37_21160, FPQ37_33410, FPQ37_01030, FPQ37_04980, FPQ37_08490	GON15_22070, GON15_25915, GON15_29285, GON15_14215, GON15_15590, GON15_16675, GON15_01930, GON15_02075, GON15_04050, GON15_05870
Quorum sensing system	bspI2	ND	ND	ND	ND	ND
	bspI3	ND	ND	ND	FPQ37_18040	ND
	bspR4	ND	NL30_36195	ND	ND	ND
	pmlI/bspI1	SK875_B00567	NL30_02710	WI95_25580	FPQ37_19250	GON15_16090
	pmlR/bspR1	SK875_B00569	NL30_02720	WI95_25570	FPQ37_19260	GON15_16080
Bsa T3SS	bapA	ND	ND	ND	ND	ND
	bicA	ND	NL30_37140	ND	ND	ND
	bprA	ND	ND	WI95_22305	FPQ37_34000, FPQ37_38855,	ND

					FPQ37_39170, FPQ37_40335, FPQ37_17425	
	bsaQ	SK875_B00413	NL30_37190	ND	ND	GON15_16865
	bsaS	ND	NL30_37180	ND	ND	ND
	bsaX	ND	NL30_37155	ND	ND	ND
	spaP	ND	NL30_37160	ND	ND	ND
T6SS-1	Undetermined	ND	ND	WI95_32775	ND	ND
	Undetermined	ND	NL30_34485	ND	ND	ND
Pyochelin receptor	fptA	SK875_B00268	ND	ND	FPQ37_32455	GON15_17585
Pyochelin	pchB	SK875_B00259	ND	ND	FPQ37_32410	GON15_17630
	pchC	SK875_B00260	ND	ND	FPQ37_32415	GON15_17625
	pchD	SK875_B00261	ND	ND	FPQ37_32420	GON15_17620
	pchE	SK875_B00263	ND	ND	FPQ37_32430	GON15_17610
	pchF	SK875_B00264	ND	ND	FPQ37_32435	GON15_17605
	pchG	SK875_B00265	ND	ND	FPQ37_32440	GON15_17600
	pchH	SK875_B00266	ND	ND	ND	GON15_17595
	pchR	SK875_B00262	ND	ND	FPQ37_32425	GON15_17615
Catalase	katA	SK875_B02015	NL30_00985	WI95_27395	FPQ37_26715	GON15_08820

Table S9. Antibiotic resistance genes in five *B. contaminans* genomes

Product	Identified strains
Catalase-peroxidase KatG (EC 1.11.1.21)	MS14, FL-1-2-30-S1-D0, ZCC, SK875, XL73
Aminoglycoside 6-phosphotransferase, putative	MS14, FL-1-2-30-S1-D0, ZCC, SK875, XL73
Class A beta-lactamase (EC 3.5.2.6)	MS14, FL-1-2-30-S1-D0, ZCC, SK875, XL73
Class C beta-lactamase (EC 3.5.2.6)	MS14, FL-1-2-30-S1-D0, ZCC, SK875, XL73
Class D beta-lactamase (EC 3.5.2.6)	MS14, FL-1-2-30-S1-D0
Alanine racemase (EC 5.1.1.1)	MS14, FL-1-2-30-S1-D0, ZCC, SK875, XL73
D-alanine--D-alanine ligase (EC 6.3.2.4)	MS14, FL-1-2-30-S1-D0, ZCC, SK875, XL73
1-deoxy-D-xylulose 5-phosphate reductoisomerase (EC 1.1.1.267)	MS14, FL-1-2-30-S1-D0, ZCC, SK875, XL73
Translation elongation factor G	MS14, FL-1-2-30-S1-D0, ZCC, SK875, XL73
Translation elongation factor Tu	MS14, FL-1-2-30-S1-D0, ZCC, SK875, XL73
Dihydrofolate reductase (EC 1.5.1.3)	MS14, FL-1-2-30-S1-D0, ZCC, SK875, XL73
Dihydropteroate synthase (EC 2.5.1.15)	MS14, FL-1-2-30-S1-D0, ZCC, SK875, XL73
DNA gyrase subunit A (EC 5.99.1.3)	MS14, FL-1-2-30-S1-D0, ZCC, SK875, XL73
DNA gyrase subunit B (EC 5.99.1.3)	MS14, FL-1-2-30-S1-D0, ZCC, SK875, XL73
Enoyl-[acyl-carrier-protein] reductase [NADH] (EC 1.3.1.9)	MS14, FL-1-2-30-S1-D0, ZCC, SK875, XL73
Isoleucyl-tRNA synthetase (EC 6.1.1.5)	MS14, FL-1-2-30-S1-D0, ZCC, SK875, XL73
3-oxoacyl-[acyl-carrier-protein] synthase, KASII (EC 2.3.1.179)	MS14, FL-1-2-30-S1-D0, ZCC, SK875, XL73
UDP-N-acetylglucosamine 1-carboxyvinyltransferase (EC 2.5.1.7)	MS14, FL-1-2-30-S1-D0, ZCC, SK875, XL73
Transcription termination factor Rho	MS14, FL-1-2-30-S1-D0, ZCC, SK875, XL73
DNA-directed RNA polymerase beta subunit (EC 2.7.7.6)	MS14, FL-1-2-30-S1-D0, ZCC, SK875, XL73
DNA-directed RNA polymerase beta' subunit (EC 2.7.7.6)	MS14, FL-1-2-30-S1-D0, ZCC, SK875, XL73
SSU ribosomal protein S10p (S20e)	MS14, FL-1-2-30-S1-D0, ZCC, SK875, XL73
Undecaprenyl-diphosphatase BcrC (EC 3.6.1.27), conveys bacitracin resistance	MS14, FL-1-2-30-S1-D0, ZCC, SK875, XL73
Enoyl-[acyl-carrier-protein] reductase [NADH] (EC 1.3.1.9), FabV => refractory to triclosan	MS14, FL-1-2-30-S1-D0, ZCC, SK875, XL73
Tetracycline resistance, MFS efflux pump => unclassified	MS14, FL-1-2-30-S1-D0, ZCC, SK875, XL73
Outer membrane factor (OMF) lipoprotein associated wth EmrAB-OMF efflux system	MS14, FL-1-2-30-S1-D0, ZCC, SK875, XL73
Multidrug efflux system EmrAB-OMF, inner-membrane proton/drug antiporter EmrB (MFS type)	MS14, FL-1-2-30-S1-D0, ZCC, SK875, XL73
Multidrug efflux system EmrAB-OMF, membrane fusion component EmrA	MS14, FL-1-2-30-S1-D0, ZCC, SK875, XL73
Macrolide-specific efflux protein MacA	MS14, FL-1-2-30-S1-D0, ZCC, SK875, XL73
Macrolide export ATP-binding/permease protein MacB	MS14, FL-1-2-30-S1-D0, ZCC, SK875, XL73
Outer membrane factor (OMF) lipoprotein associated wth MdtABC efflux system	MS14, FL-1-2-30-S1-D0, ZCC, SK875, XL73
Multidrug efflux system MdtABC-TolC, inner-membrane proton/drug antiporter MdtB (RND type)	MS14, FL-1-2-30-S1-D0, ZCC, SK875, XL73

Multidrug efflux system MdtABC-TolC, inner-membrane proton/drug antiporter MdtC (RND type)	MS14, FL-1-2-30-S1-D0, ZCC, SK875, XL73
Multidrug efflux system MdtABC-TolC, membrane fusion component MdtA	MS14, FL-1-2-30-S1-D0, ZCC, SK875, XL73
Multidrug efflux system, inner membrane proton/drug antiporter (RND type) => MexY of MexXY/AxyXY	MS14, FL-1-2-30-S1-D0, ZCC, SK875, XL73
Multidrug efflux system, membrane fusion component => MexX of of MexXY/AxyXY	MS14, FL-1-2-30-S1-D0, ZCC, SK875, XL73
Multidrug efflux system, outer membrane factor associated with MexXY/AxyXY system	MS14, FL-1-2-30-S1-D0, ZCC, SK875, XL73
16S rRNA (guanine(527)-N(7))-methyltransferase (EC 2.1.1.170)	MS14, FL-1-2-30-S1-D0, ZCC, SK875, XL73
Glycerophosphoryl diester phosphodiesterase (EC 3.1.4.46)	MS14, FL-1-2-30-S1-D0, ZCC, SK875, XL73
CDP-diacylglycerol--glycerol-3-phosphate 3-phosphatidyltransferase (EC 2.7.8.5)	MS14, FL-1-2-30-S1-D0, ZCC, SK875, XL73
Outer membrane low permeability porin, OprB family	MS14, FL-1-2-30-S1-D0, ZCC, SK875, XL73
Outer membrane low permeability porin, OprD family	MS14, FL-1-2-30-S1-D0, ZCC, SK875, XL73
DNA-binding protein H-NS	MS14, FL-1-2-30-S1-D0, ZCC, SK875, XL73
Hydrogen peroxide-inducible genes activator => OxyR	MS14, FL-1-2-30-S1-D0, ZCC, SK875, XL73
